# Effect of Spanish-Style Table Olive Processing on Fatty Acid Profile: A Compositional Data Analysis (CoDA) Approach

**DOI:** 10.3390/foods11244024

**Published:** 2022-12-13

**Authors:** Antonio Garrido-Fernández, Amparo Cortés-Delgado, Antonio López-López

**Affiliations:** Instituto de la Grasa (CSIC), Food Biotechnology Department, Campus Universitario Pablo de Olavide, Edificio 46. Ctra. Utrera, km 1, 41013 Sevilla, Spain

**Keywords:** compositional data analysis, Manzanilla cv, Hojiblanca cv, fatty acids, Spanish-style green table olives, processing

## Abstract

This manuscript considers that the composition of Manzanilla and Hojiblanca fats are compositional data (CoDa). Thus, the work applies CoDa analysis (CoDA) to investigate the effect of processing and packaging on the fatty acid profiles of these cultivars. To this aim, the values of the fat components in percentages were successively subjected to exploratory CoDA tools and, later, transformed into *ilr* (isometric log-ratio) *coordinates* in the Euclidean space, where they were subjected to the standard multivariate techniques. The results from the first approach (bar plots of geometric means, tetrahedral plots, compositional biplots, and balance dendrograms) showed that the effect of processing was limited while most of the variability among the fatty acid (FA) profiles was due to cultivars. The application of the standard multivariate methods (i.e., Canonical variates, Linear Discriminant Analysis (LDA), ANOVA/MANOVA with bootstrapping and *n* = 1000, and nested General Linear Model (GLM)) to the *ilr coordinates* transformed data, following Ward’s clustering or descending order of variances criteria, showed similar effects to the exploratory analysis but also showed that Hojiblanca was more sensitive to fat modifications than Manzanilla. On the contrary, associating GLM changes in *ilr* with fatty acids was not straightforward because of the complex deduction of some *coordinates*. Therefore, according to the CoDA, table olive fatty acid profiles are scarcely affected by Spanish-style processing compared with the differences between cultivars. This work has demonstrated that CoDA could be successfully applied to study the fatty acid profiles of olive fat and olive oils and may represent a model for the statistical analysis of other fats, with the advantage of applying appropriate statistical techniques and preventing misinterpretations.

## 1. Introduction

Over the last decades, a comprehensive study of Spanish table olive cultivars’ fatty acid profiles was undertaken by López et al. [1]. In green Spanish-style table olives, the proportion of fat in the edible flesh ranged from 11 (Gordal) to 16 (Manzanilla) g/100 g wet flesh. Their major fatty acid components were C18:1c, C16:0, C18:2n-6, and C18:0 [1], in proportions comparable to those reported for olive oil [2].

Usually, the fatty acid profile of olive oil is estimated, according to Commission Regulation (ECC) No 2568/91 [3], by:$\;w_{i}=(A_{i}/\sum A)\times 100$ where A_i_ is the area under the peak of each fatty acid (FA) methyl ester (i) and ∑A is the sum of the areas of all FA peaks. The composition of olive oil in FAs is then expressed as the percentages by mass of their corresponding methyl esters. They are always characterised by being positive and sum to a constant value, usually 100.

According to the literature, these values are compositional data (CoDa) [4] and belong to the simplex metric space [5], where they should be represented and interpreted. Thus, the standard multivariate methods developed for the Euclidean space should not be applied to them [6]. Among the reasons that Van den Boogart and Tolosana-Delgado [7] mention for considering inappropriate the application of the standard multivariate statistics, developed for real-value data sets, to compositions are: (i) individual components mixed and closed exhibit negative correlations, which contradicts the usual interpretation of correlations and covariance; (ii) correlations and covariance between two parts depend on the components included in the analysis; (iii) due to the row (cases) constant sum, variance matrices are singular; (iv) the bounded range of values also implies that components cannot be normally distributed.

Recent studies on the overall fat composition of the most relevant cultivars devoted to table olive revealed that several FA proportions in their fats did not agree with the limits established for the characteristics of the olive oil categories [8,9,10,11,12]. Furthermore, marked differences are also observed between the limits established by the IOC/EU and other international regulations [13]. In addition, the percentages of components depended on the number of FAs considered in the analysis. On the contrary, the application of partial CoDa analysis (CoDA) to the fatty acid profile of Spanish-style Gordal [8], pig fat [14], or a preliminary study of a reduced set of these data [15] was successful. Recent advances and publications on CoDA [16,17] make the application of the new statistical tools to table olive fatty acid profiles challenging. This work represents a simple approach to studying them exclusively with CoDA.

There is a general concern about the relationship between health and food [18]. Many governments have developed legislation to improve information on food nutrients [19,20]. Table olives have been essential in the Mediterranean diet, but they are currently consumed worldwide, reaching a production of about 3·10^6^ tonnes for the 2020/2021 season [21]. The green Spanish-style table olives enjoy consumers’ preference because of their attractive aspect, organoleptic characteristics, and numerous presentations. Their processing includes lye treatment, washing with tap water, brining, fermentation, and packaging, which produce marked transformations in the fruits’ physicochemical characteristics. Their primary nutrient is fat [22]. Therefore, studying the modifications in the fatty acid profile with the appropriate statistical tools seems a reasonable challenge.

This manuscript considers that the composition of Manzanilla and Hojiblanca fats are compositional data (CoDa). Thus, the work applies CoDa analysis (CoDA) to investigate the effect of processing and packaging on the fatty acid profiles of these cultivars. To this aim, the values of the fat components in percentages were successively subjected to exploratory CoDA tools and, later, transformed into *ilr* (isometric log-ratio) *coordinates* in the Euclidean space, where they were subjected to the standard multivariate techniques [6,16,17,23]. Since the standard multivariate techniques were developed for this space, such coordinates were subjected to them without any restriction. The results of this study would demonstrate the possibility of using CoDA for the statistical analysis of fat compositions in general.

## 2. Materials and Methods

### 2.1. Cultivars

The raw material was hand-picked olives of the Manzanilla and Hojiblanca cultivars, at the so-called green maturation stage (Maturity Index = 1, according to Ferreira [24]), provided by a local processor (JOLCA S.L., Huevar, Sevilla, Spain). The fruits were subjected to processing 24 h after harvesting.

### 2.2. Processing 

The olives were processed according to the green Spanish style [22]. The fresh olives were treated with 2.5 and 3.0 g/100 mL NaOH solutions for Manzanilla (M) and Hojiblanca (H), respectively, up to 2/3 flesh (≈7 h). Then, olives were submerged in tap water for 18 h and brined in a 9 g NaCl/100 mL solution. After seven months of spontaneous fermentation, the olives were packed and pasteurised (80 °C for 15 min). The experiment was run in duplicate. Two samples from the fresh fruits of each cultivar (T0), as well as from each replicate of the lye-treated olives (T1), the fermented fruits (T2), and two-months-packaged fruits (T3) were successively withdrawn. They were coded as MT0, MT1, MT2, and MT3 and HT0, HT1, HT2, and HT3.

### 2.3. Oil Extraction

The fruits were ground using an Ultraturrax T25 (IKA-Labortecnik, Staufen, Deutschland), and the oil was removed by centrifugation in ABENCOR equipment. The method is fully described elsewhere [25,26]. Because of the mild extraction conditions, the procedure causes minimal changes in the oil [8].

### 2.4. Fatty Acid Composition

The method for analysing the fatty acid profiles is described elsewhere [25,26]. 

The methyl esters were determined using a Hewlett-Packard 5890 series II gas chromatograph, a fused silica capillary column Select FAME (100 m × 0.25 mm, 0.25 μm film thickness) (Varian, Bellafonte, PA, USA), and a flame ionisation detector. Quantification was achieved according to Commission Regulation (ECC) No 2568/91 [3]. Values are the average of two determinations per sample.

### 2.5. Statistical Analysis

Fatty acid profiles of olive oil or table olive fats are habitually studied by standard multivariate statistical tools. However, according to Aitchison [4], they represent a straightforward case of compositional data which contains only relative information. The techniques used for their analysis must respect their scale properties [17] and the new CoDa geometrical and statistical approach. The CoDA tools applied were bar plots of geometric means, tetrahedral plots, compositional biplot, balance dendrogram, and *ilr* transformation of the original values into *coordinates* in the Euclidean space (to apply standard multivariate techniques). Appendix A includes a succinct description of them. Some brief explanations are also included in the next text sections when appropriate. 

For data processing and graph drawing, the programs used were Statistica software version 8.0 (StatSoft Inc., Tulsa, OK, USA), the plugin XLSTAT v.2017 (Addinsoft, Paris, France, v. 2017), CoDaPack v 2.03.01 [27], and robCompositions [28] run in R (v 4.0.3) [29], under RStudio (v 1.4.1103) [30].

## 3. Results and Discussion

The physicochemical characteristics of brines after fermentation were: pH~4.0; titratable acidity, ~1 g lactic acid/100 mL; combined acidity, ~0.12 Eq/L; NaCl level, ~6.0 g/100 mL. Equilibrium conditions in brine after packaging were 5 g lactic acid/100 mL and 5.5 g NaCl/100 mL. These parameters’ levels are typical for green Spanish-style fermentation and commercial products, respectively [22].

### 3.1. Fatty Acid Data Set

The matrix of data comprised 28 rows (*i*), four for the raw material (two cultivars × two samples each) + 24 for the processing phases (two cultivars × three processing phases × two replicates × two samples), and 20 columns (*j*) (one for the names of the observations (samples) and 19 for the quantified fatty acids) (Table S1). Each cell x*_ij_* was the average fatty acid value of two analyses per sample.

First, verifying the conditions that justify the improper application of conventional standard statistics to table olive (or olive oil) fatty acid profiles is intended. If present, the studies applying them might lead to misleading conclusions. In agreement with the first reason (i) stated in the Introduction section, the initial data set (expressed as percentages) led to multiple negative correlations and covariance values between the same pairs of FAs in both the complete set and sub-compositions. For example, see results from only those more relevant fatty acids C16:0, C18:0, C16:1, C18:1c, and C18:2n-6, representing 97.57% of the total variance (Table 1 and Table 2). Moreover, as expected from argument (ii), the correlations and covariance values depended on the components in the sub-composition (Table 1 and Table 2). Finally, reasons (iii) and (iv) are present in the usual FA data sets just because of their structure (constant row sums). Therefore, applying standard multivariate methods to the habitual table olive (and olive oil) FA profiles suffers from the drawbacks mentioned by Van den Boogart and Tolosana-Delgado [7] and the analysis of table olive fat by CoDA techniques is not optional but an appropriate statistical approach.

### 3.2. Evaluation of Group Trends by Geometric Mean Bar Plots

The geometric mean is recommended as a proper alternative to the standard mean for describing the central tendency of data in the simplex or Aitchison geometry [5]. It can also give an overview of the effect of treatments on compositions [16]. The most abundant FAs in olive fat were C18:1c, C16:0, C18:2n-6, and C18:0 (Table 3), with similar proportions as in olive oil [2] or green Gordal table olive fat [8]. The geometric mean of C16:0 was higher in Manzanilla than in Hojiblanca (Table 3). An opposed relationship was observed for C18:1c. The remaining comparisons between the geometric mean contents in the two cultivars are similarly deduced from Table 3.

**Figure 1:**
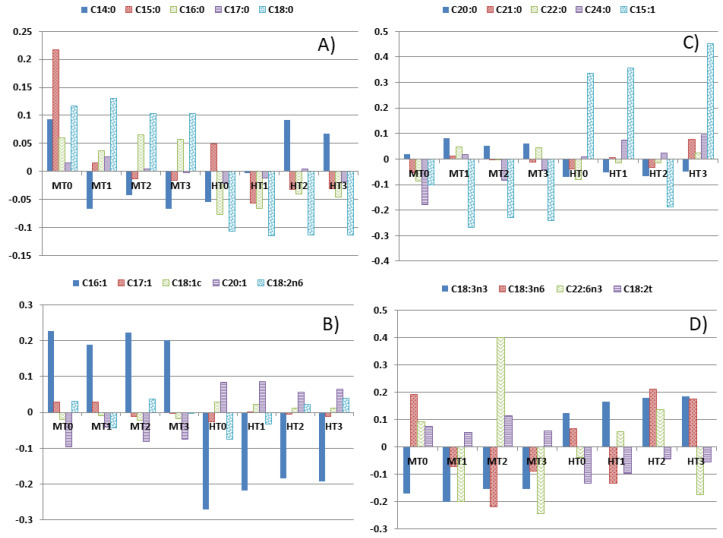
Geometric mean bar plots (relative to the overall geometric mean) for the green Spanish-style table olive according to cultivars and processing phases. (**A**–**D**) are for the study of different groups of the analysed fatty acids. M, Manzanilla; H, Hojiblanca; T0, fresh olives; T1, lye-treated olives; T2, fermented olives; T3, packaged olives. Treatments within cultivar are formed by combining cultivar and processing phases.

The plot of the log-ratio (that is, the difference between their natural logs) of each FA content (geometric mean), according to processing phases and cultivar, over the overall geometric mean is an approach for estimating the effects of such variables (Figure 1). The graph shows that C16:0 (mainly) or C18:0 (Figure 1A) and C20:0 or C16:1 (mostly) (Figure 1C) are more abundant in Manzanilla (positive log-ratios) than in Hojiblanca (negative). On the contrary, C15:1 (mostly) (Figure 1B), C20:1 (Figure 1C), and C18:3n-3 (Figure 1D) were more abundant in Hojiblanca. Differences in percentages between cultivars were previously reported for C18:0, C20:0, and C16:1 (higher in Manzanilla) as well as for C18:1c, C18:3n-3, and C22:6n-3 (higher in Hojiblanca) but not for C16:0, C15:1, or C20:1 [3]. Regarding processing phases (within cultivar), the changes noticed were always observable but less pronounced than those appreciated between cultivars: e.g., C14:0 or C15:0 in Manzanilla (Figure 1A) or C16:1 in Hojiblanca (Figure 1C). Moreover, the log-ratio values (Table 3) could also be subjected to standard statistical tests. The bar plot discloses the changes intuitively but depends on the entire set of variables through the overall geometric means. This circumstance is a clear limitation and makes necessary to complement this information with other statistical techniques.

### 3.3. Study of Log-Ratio Dispersion through the Variation Array Matrix

In CoDA, there is no counterpart to standard deviation directly constructed from the original composition. Instead, considering that compositional data only carry relative information, the approach for measuring dispersion focuses on pairwise log-ratios, with their variances gathered in the so-called variation array [4]. The overall variance of a specific part is deduced from the row-wise log-ratios of each component over the other parts (Table 4, upper half diagonal) or from its *clr coefficients* (Table 4, last column on the right). The value for each part is the so-called *clr* variance, which includes the variance due to differences between groups and other sources. The *clr* variance is sometimes the criterion for selecting the variables used for checking the effects of the design factors [31,32]. The greatest *clr* variance value was for C15:1, followed by C18:3n-6, C22:6n-3, C16:1, C18:3n-3, C18:0, C24:0, C18:2t, C15:0, C20:0, C20:1, C21:0, C16:0, and so on (Table 4, last column on the right). In the order mentioned, they could then be candidates for sequential binary partition (SBP) since the first balances (*ilr coordinates*) may explain the greatest variance proportions. Then, these usually decrease as *clr* variances are lower.

The variation array is symmetric regarding its diagonal, so the lower half is substituted with the corresponding log-ratios to provide additional information. These values were initially used for standard multivariate analysis, but nowadays, other transformations with better mathematical properties (e.g., *ilr coordinates*) are preferred.

### 3.4. Tetrahedral Display

The triangle and tetrahedral plots are the simplest form of CoDa representation in the Aitchison geometry (or simplex). They show the cases as a function of three and four parts, respectively [33]. The lower the abundance of a part is, the closer the sample is to the opposing border (face) to its vertex. Usually, centring the data before plotting prevents grouping of the samples close to faces or vertices. These plots can offer a rough image of the data distribution and groups’ trends [17]. Compositions with numerous parts may be plotted as a function of those with the highest variance and expected greater segregation power. Here, C15:1, C18:3n-6, C22:6n-3, and C16:1 (with the most relevant *clr* variance in the variation array) were chosen (Figure S1). Notice that the centred samples from the two cultivars, regardless of the processing phase, are fairly well segregated. However, no trend within cultivars is observed, in agreement with Figure 1, where differences between cultivars were apparent but no effect of processing phases was clear. No linear trend along Principal Components (PCs) was noticed either. In Gordal olives [8], the FAs with the greatest variance were C14:0, C18:2t, C18:3n-6, and C:22:6n-3 and the tetrahedral graph of samples as a function of them showed that the FA profile of the fresh fruits was affected by processing. Besides, the extraction by *Soxhlet* significantly affected the FA profiles compared to the method using Abencor. Notice that C18:3n-6 and C22:6n-3 showed high variability in this work and Gordal experiments [8].

### 3.5. Compositional Biplot

**Figure 2:**
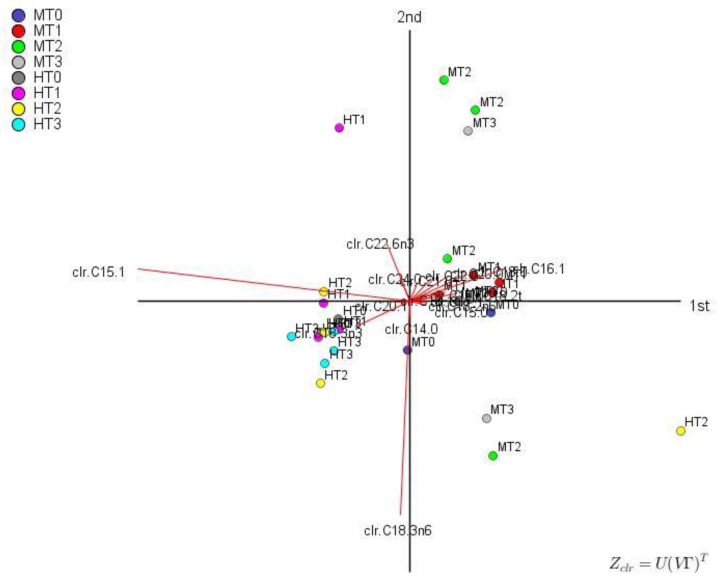
CoDa covariance biplot, appropriate to study the relationship among variables. PC1 accounted for 45.70% variance and PC2 for 26.07% (together, 71.77%). M, Manzanilla; H, Hojiblanca; T0, fresh olives; T1, lye-treated olives; T2, fermented olives; T3, packaged olives.

The adaptation of the traditional biplot to compositional data, based on *clr coefficients*, was developed by Aitchison and Greenacre [34] and is usually drawn in two dimensions. On it, row and column points are both centred at the origin of the display. Depending on the factorisation, two main types are obtained: (i) covariance biplot (*ν* = 0), preserving covariance structure between log-ratios (Figure 2), and (ii) form biplot (*ν* = 1), which preserves distances between observation (rows) (Figure S2). Following Aitchison and Greenacre [34], the distances between the ends of two rays (parts) in the covariance biplot approximate the standard deviation of their log-ratios (Figure 2); the highest values are between C15:1 and C16:1 (and the other FAs in the first quadrant). As well, log-ratios of C18:3n-6 over C22:6n-3, C16:1 or practically any other FA have large standard deviations (Figure 2). These log-ratios thus have good potential segregation power between processing phases. Moreover, the cosine between two links could be an estimation of the correlation between the two log-ratios; thus, ln(C15:1/C16:1) and ln(C18:3n-6/C22:6n-3) are not related at all since their links form an angle of ~90° (whose cosine is about 0), but ln(C18:3n-6/C22:6n-3) and ln(C18:3n-6/C14:0) are closely associated since their angle is low (Figure 2). Thus, the covariance biplot resulted in an interesting tool to identify the log-ratios with considerable variation between groups.

In the form biplot (Figure S2), the separation between individuals is an approximations of distances between observations (rows). There is segregation between cultivars (Hojiblanca on the left and Manzanilla on the right), based mainly on PC1 (strongly associated with C15:1 (negatively), C16:1 (positively), and many other FAs); however, there was no clear segregation among processing phases, in agreement to the previous results. PC2 (linked negatively to C18:3n-6 and positively to C22:6n-3) had low differentiation power.

Differences in FA compositions have been reported for Turkish cultivars, allowing the segregation of Hurma cultivar from Erkence and Gemlik cultivars [35]. Issaoui et al. [36] studied the autochthonous Meski, Picholine, and Manzanilla cultivars according to two Tunisian processes, observing significant differences among cultivars but not an effect of processing. However, they applied standard multivariate statistics. Sensible differences between cultivars were also observed among the oil-fatty compositions of eighteen Mediterranean cultivars cultivated under the arid conditions of Boughrara in Tunisia [9].

### 3.6. Application of the Sequential Binary Partition for Deducing ilr Coordinates

As mentioned by diverse authors [6,16,17,23], the transformation of the original data set into *ilr coordinates* in the Euclidean space is a necessary step for applying standard multivariate techniques in CoDA since it moves the values into the Euclidean space (see brief explanation in Supplementary Material). There are no specific criteria for selecting the fatty combinations to construct *ilr coordinates*. Ideally, the procedures chosen should lead to the maximum segregation power when evaluating the effects of variables. In this work, they were based on the decreasing order of *clr* variances and Ward’s clustering.

#### 3.6.1. Sequential Binary Partition Following the Decreasing Order of *clr* Variances

The SBP was applied following the formula provided in Appendix A. It was expected that the best segregation between observations, using the decreasing order of *clr* variance, would include in the first *ilr coordinate*, apart from the normalisation parameter, the log-ratio between the fatty acid with the greatest *clr* variance (C15:1) and the geometric mean of the rest:

$$\mathrm{ilr}1=\sqrt{\left(\frac{1*18}{1+18}\right)}\times \lfloor ln\;\frac{C15\text{:}1}{{\left(C14\text{:}0\cdot C15\text{:}0\cdot \ldots C22\text{:}6n3\cdot C18\text{:}2t\right)}^{1/18}}\rfloor$$

Following a similar criterion, the second was:

$$\mathrm{ilr}2=\sqrt{\frac{1*17}{1+17}\;}\times \lfloor ln\;\frac{C18\text{:}3n6}{{\left(C14\text{:}0\cdot C15\text{:}0\cdot \ldots C22\text{:}6n3\cdot C18\text{:}2t\right)}^{1/17}}\rfloor$$

where C15:1, already used in *ilr*1, is removed from the calculation not only of *ilr*2 but also of the subsequent coordinates. The decreasing order of variances criterion was applied in the first 13 balances. However, after this point, the SBP followed the successive unused FA over the remaining ones because of the similarity of their variances. The operation was repeated row-wise for all the samples of each cultivar-processing phase combination. The SBP is summarised in a matrix where the FA is identified, row-wise, by 1, −1, or 0 when the value in the initial data set is included in the numerator, denominator, or does not take part in the partition, respectively (e.g., C15:1 shows 1 for the first balance and 0 for the rest) (Table S2). This matrix allows reproduction of the SBP and is also helpful for back-transformation (*ilr coordinates* into the original scale). In this way, the actual fatty acid composition matrix (D parts) is substituted row-wise with its D-1 *ilr coordinate* matrix (Table S3) since the last partition combines the cells of the last two columns in just one. The data defined by the SBP matrix represent a set of *ilr coordinates* in orthonormal axes (basis) that can be subjected to the standard multivariate techniques. However, it has previously been observed that *clr* variance may be caused not only by the effect of treatments but also by determination errors in cases of low contents [8,14]. Therefore, checking alternative criteria is important.

#### 3.6.2. Use of Cluster Analysis for Sequential Binary Partition Selection

**Figure 3:**
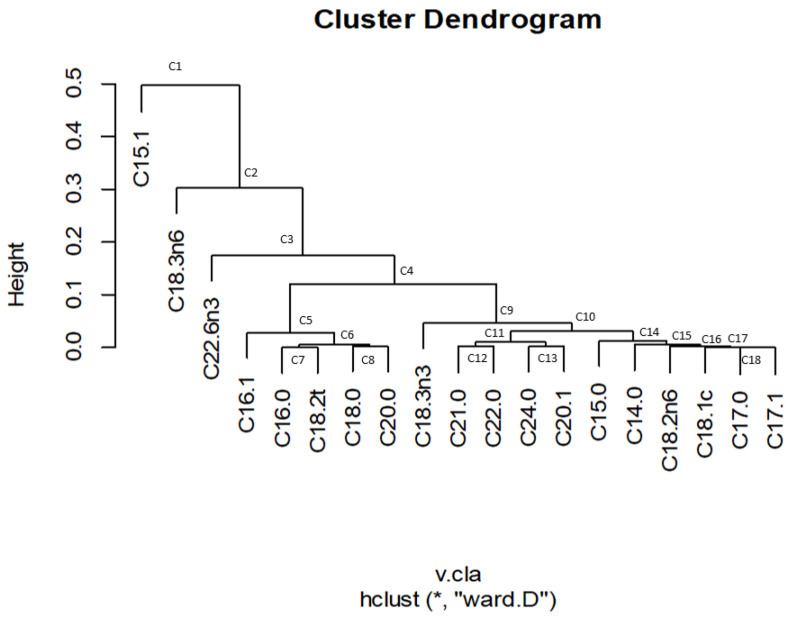
Clustering analysis of FAs (*, Q-Mode), using Ward’s method. This segregation was also one of the criteria used for selecting the FAs’ order in the SBP of the original data set.

Clustering parts in CoDA face the problem of choosing the measure for distances or dissimilarities. The variation matrix is usually the proper data set [17]. The Ward’s clustering method (Figure 3) minimises the within-cluster variance [17]. When the SBP followed the clustering order, the decreasing heights were successively observed for C15:1 over the remaining fatty acids, C18:3n-6 over their remaining fatty acids, and C22:6n-3 over the still remaining fatty acids. Therefore, these log-ratios could be chosen for the first three steps of *ilr coordinates* and could provide the most remarkable segregation power of observations. In addition, these *ilr coordinates* were similar to those selected using the decreasing order of *clr* variances. However, the following partitions corresponded to groups of FAs. Thus, in the next log-ratio, C16:1, C16:0, C18:2t, C18:0, and C20:0 were assigned to the numerator and the remaining ones on its right to the denominator. Similar binary partitions could be deduced attending the successive cluster sequences (Figure 3), which confront single or groups of FAs within the following clusters of two groups. As observed in Figure 3, most of the consecutive steps included several FAs until ending with opposing only two FAs. As previously, the SBP is summarised in a matrix (Table S4), which identifies, row-wise, by 1, −1, or 0, when the value in the initial data set is included in the numerator, denominator, or does not take part in the partition, respectively. As before, this matrix allows reproduction of the SBP and the back-transformation (*ilr coordinates* into the original scale). The resulting *ilr coordinates* matrix is presented in Table S5. As previously mentioned, their values are on an orthonormal basis, and the means and variances of the resulting data set can be obtained by the usual procedures (Table S6, for overall estimations). Moreover, this data set can also be analysed using standard statistics [6,16,17,23]. The values obtained using the *clr* decreasing variance and Ward’s criteria differ (Table S6) because of their different basis, but their total variance (0.607) and conclusions after applying the standard multivariate techniques to both are the same. Also, both reproduce the original data structure.

### 3.7. Balance Display by the Compositional Dendrogram

**Figure 4:**
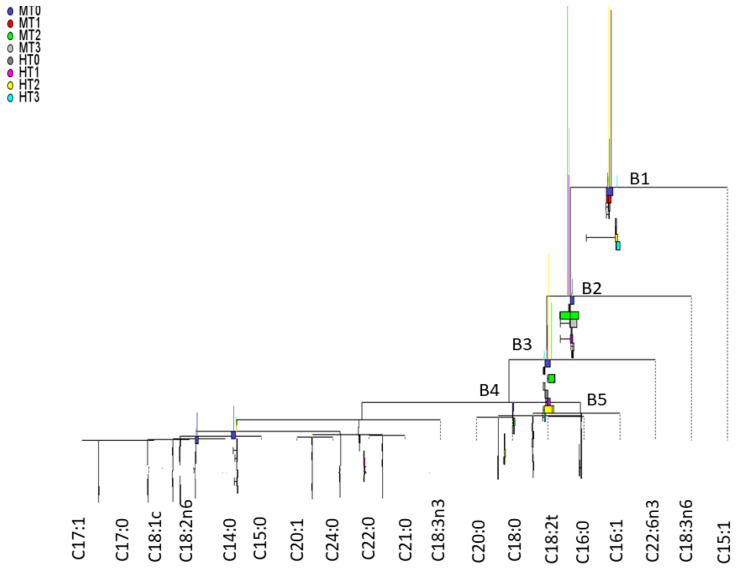
Balance dendrogram, based on the sequential binary partition of parts according to Ward’s clustering ordination. M, Manzanilla; H, Hojiblanca; T0, fresh olives; T1, lye-treated olives; T2, fermented olives; T3, packaged olives. Only the first relevant balances are enumerated.

The CoDa dendrogram is the graphical presentation of balances and variances [6,23]. To display the effects of the factors, the balances were estimated based on overall averages and according to groups (cultivars and processing phases). In the plots (Figure 4 and Figure S3), the overall balance is represented on the horizontal axis. Those corresponding to groups are displayed as boxes situated below these axes. A displacement towards the right (left) indicates that the geometric mean of parts in the SBP numerator predominates over those in the denominator (or vice versa). A centred position means equilibrium between them. The vertical lines stand for the variances of the corresponding balances. The plot allows the comparison of the overall balances (and their variances) as well as the contrasts among the levels of factors (and their variances). The first three balances (B1–B3) are displaced toward the left (negative log-ratios) (Figure 4 and Figure S3), signifying an overall lower presence of C15:1, C18:3n-6, and C22:6n-3 over the geometric means of the remaining FAs. However, the segregation power between cultivar and processing phase was limited (scarce displacements of boxes below the horizontal axes) (Figure 4 and Figure S3).

Regarding variances (vertical lines), those corresponding to the three first balances are the most relevant irrespective of the criterion used for the SBP estimation. Under them, the balances of the processing steps of Hojiblanca and Manzanilla are visible, although their comparison is difficult because of the numerous factor levels. For variances according to groups, those from MT2 and HT2 (fermented olives) showed the largest values. An association between low contents with large variances is generally apparent, as already pointed out in the literature [37]. The remaining balances showed alternant signs and reduced variances (Figure 4 and Figure S3). Thus, the effects of cultivar and processing phases on most FAs were relatively low.

So, the two criteria followed to define the SBP led to dissimilar CoDa dendrograms (only sharing the three first balances) with different averages and variances because of the diverse basis. The basis used to determine the *ilr coordinates* were just two of the possible options, but there is no guarantee that any other non-tested were more appropriate for describing the effects of cultivars and processing phases. However, they will explain the same overall and total *clr* variances (Table 4). Regardless of the criterion, the C15:1 was the FA of Manzanilla and Hojiblanca with the highest variance in its balance, while C14:0 was in Gordal [8]. This may indicate fatty acid profile structural differences among cultivars.

### 3.8. Application of the Standard Multivariate Statistical Tools to Coordinates

#### 3.8.1. Canonical Variates Plot and Linear Discriminant Analysis

A canonical variate is a new variable obtained as a linear combination of the original ones attempting to distinguish between groups. As Martin et al. [16] state, a canonical variate in CoDA is

$$y=v^{T}ilr\left(x\right)=\sum_{j=1}^{D-1}v_{k}ilr{\left(x\right)}_{k},\;k=1,\text{…},\;D$$

where y is a log-contrast. In this technique, the first canonical variate is defined by the vector $v_{1}$ which maximizes the F value associated with the ANOVA test:

$$H0\text{:}\;v_{1}^{T}\cdot \mu_{1}=\ldots =v_{1}^{T}\mu_{g},\;\mu_{j}=1,\ldots ,\;g\;\left(\mathrm{centres}\;\mathrm{of}\;\mathrm{the}\;\mathrm{groups}\right)$$

**Figure 5:**
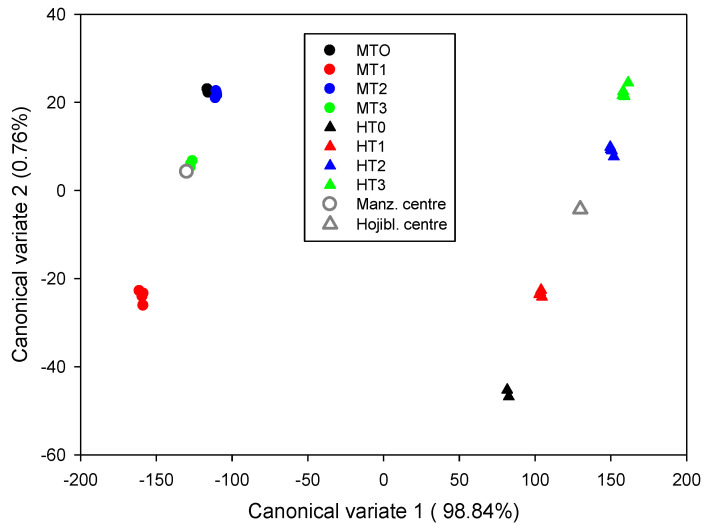
Segregation of processing phases by canonical variate plot of *ilr coordinates*, according to cultivars. The *coordinates* were obtained following Ward’s clustering structure. The overall centres for Manzanilla and Hojiblanca are also plotted. M, Manzanilla; H, Hojiblanca; T0, fresh olives; T1, lye-treated olives; T2, fermented olives; T3, packaged olives.

It can be proved that the vector $v_{1}$ is the eigenvector of the matrix $W^{-1}B\;$ associated with its maximum eigenvalue, where *W* is the within-group and *B* the between-groups sums of squares, verifying these matrices’ variability decomposition property (*T* (total) = *B* + *W*). Following this process iteratively, the *D* − 1 eigenvectors that define the corresponding canonical variates can be obtained. Importantly, if the procedure is applied to *coordinates* using a different basis, the same canonical coordinate is obtained; that is, the canonical covariate is invariant to the change of basis. Since the *coordinates* on their respective basis were different, the scores and the proportion of variances accounted for by the axes in both cases are different. Regardless of the *ilr coordinates*, there was a net separation between cultivars (Figure 5 and Figure S4), which responded differently to the treatments. In Hojiblanca, the fresh fruits (HT0) diverged from the treated ones, and, as the process progressed, they were increasingly differentiated from the raw material. In Manzanilla, the composition of the fresh product was comparable to the fermented fruits, while the lye-treated olives had the most different composition.

Principal Component Analysis (PCA) applied to Gordal table olive FA compositions expressed as percentages led to slightly different results than using *ilr coordinates* [8]. The biplot of the FAs of *Aloreña de Málaga* based on *ilr coordinates* produced clearer segregation between fresh and fresh green packaged olives with respect to stored and packaged products [38]. These results, however, deserve attention since the *ilr coordinates* reproduce the original structure of the initial data [6]. Also, the standard chemometric, based on percentages, showed non-conclusive results when analysing the effect of ripe olive processing (darkening phases) on the oil composition but detected differences among cultivars [25]. Moreover, PCA based on FA proportions properly segregated naturally debittered Turkish cultivars [35]. Apparently, in the case of notorious differences in the FA compositions (e.g., between cultivars), the standard statistics applied to data expressed in percentages may lead to about the same results to CoDA, but, at least in this case, the canonical variate plot, based on *ilr coordinates*, was somewhat more efficient to segregate changes due to processing phases.

Moreover, the *ilr coordinates* obtained by SBP based on the two options (decreasing *clr* variance and Ward’s clustering) were subjected to standard linear discriminant analysis (LDA) using bootstrapping (1000). There was 100% success in the correct assignations of the samples (Table S7); however, the results were less satisfactory in the one-out validation (below in parenthesis). Overall, *ilr coordinates* deduced following Ward’s criterion tended to have better assignations than those using the decreasing order of *clr* variance (Table S7) and could be preferable. The initial correct assignations in this work was higher than in the segregation between season, montanera length, or sampling location in Iberian pigs [14], but not in the cross-validation results, possibly because of the higher number of observations in it.

#### 3.8.2. Study of the Effects by ANOVA/MANOVA Contrasts

The one-way ANOVA tests (bootstrapping = 1000) showed no significant differences between groups (irrespective of the estimation choice) for *ilr*1 and *ilr*2 as well as for *ilr*11, *ilr*6c, *ilr*7c, *ilr*10c, *ilr*13c, and *ilr*18c (c stands for *ilr* deduced following Ward’s clustering; otherwise, from decreasing variance) (Table 5). Notice that the *ilr coordinates* from the two fatty acids with the most considerable *clr* variances and forming separated Ward’s clusters do not show significant differences in processing phases. Their large variability in the log-ratios with these FAs in their numerator was more due to low contents and subsequent significant relative errors than to effects of treatments. This analysis was a tool to detect such circumstances and prevent improper conclusions based only on the variances of their log-ratio variances.

According to Martín-Fernández et al. [16], *considering X_k_ as a random composition of a group k, where k = 1, 2,…, g, a basic model may be generated considering that X_k_ is obtained by adding a random variability ϵ_k_ around a centre μ_k_ in a multiplicative pairwise: X_k_ = μ_kʘ_ϵ_k_. In this case, the expected value of variability is 1, and the model is equivalent to ilr(X_k_) = ilr(μ_k_) + ilr(ϵ_k_), where ilr(ϵ_k_) is centred at the origin of coordinates. That is, working in coordinates, one can assume the same model as for interval scale data in the real space.* Therefore, *ilr coordinates* were subjected to MANOVA (bootstrapping = 1000), resulting in significant (*p* < 0.0001) diverse multivariate tests (Wilks, Hotelling–Lawley, Pillai, and Roy), regardless of the criterion used for the *ilr* definition. Then, the effects of cultivar and processing phases (nested in cultivar because of the differences in behaviour observed for each cultivar in the canonical analysis) were checked by GLM, using the two *ilr* estimation options.

Focusing on the significantly affected *ilr coordinates*, two *ilr* groups were observed: (i) those not (or scarcely) influenced by processing phases but showing significant differences between cultivars, and (ii) those affected by the processing phases in one or both cultivars. Because of the large number of significant *ilr*, only a few of them are commented. Regarding (i) (Figure S5), the *ilr*16, *ilr*12, and *ilr*10 were greater for Manzanilla than for Hojiblanca but reversed in the case of *ilr*5. To relate such trends with the FA profiles, one should consider the respective *ilr* definitions. For *ilr*16 (C17:1 over the geometric mean of C18:1c, C20:1, and C18:2n-6), C18:1c and C20:1 had a higher value in Hojiblanca than in Manzanilla, but similar levels of the other two FAs in both cultivars, in agreement with Figure 1C. As C18:1c and C20:1 are included in the denominator of *ilr*16, they are responsible for the lower *ilr*16 values in Hojiblanca than in Manzanilla (Figure S5A). A similar strategy can relate the remaining *ilr* changes (Figure S5) to their involved FAs.

**Figure 6:**
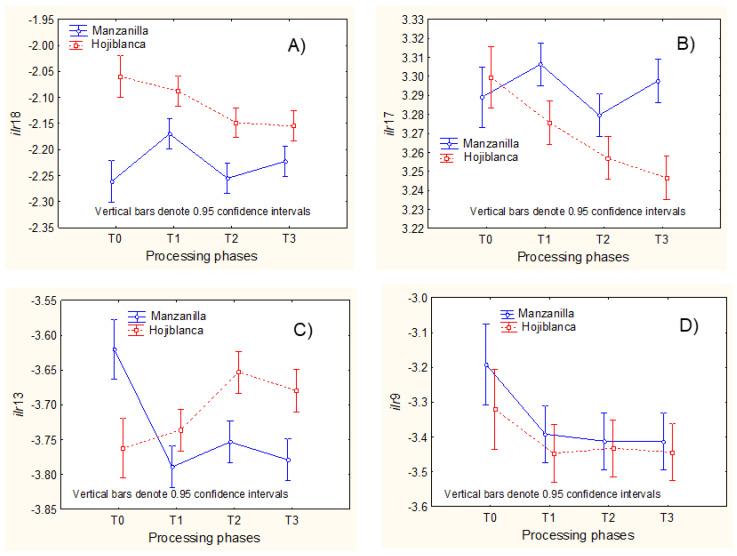
Examples of processing phase effects, according to cultivar, on *ilr coordinates* deduced from SBP using decreasing *clr* variance order. (**A**–**D**) show *ilr coordinates* which showed significant differences between cultivars. T0, fresh olives; T1, lye-treated olives; T2, fermented olives; T3, packaged olives.

Regarding the (ii) cases (Figure 6), some *ilr* estimation formulas are simple, but the explanations are not always obvious.

In *ilr*18 (log-ratio C20:1/C18:2n-6) and *ilr*17 (log-ratio C18:1c over the geometric mean of C20:1 and C18:2n-6), the decrease in Hojiblanca is probably more linked to a marked reduction in C20:1 (Figure 6A) and C18:1c (Figure 6B) than to an increase in C18:2n-6 (formation not reliable), present in both *ilr*18 and *ilr*17 denominators. Besides, the changes in C18:1c should be greater than in C20:1 (also in the *ilr17* denominator) since the decrease in *ilr*17 is more pronounced than in *ilr*18. Sometimes, although the estimation formula could be complex (e.g., *ilr*9, which is the log-ratio of C15:0 over the geometric mean of C14:0, C16:0, C17:0, C20:0, C22:0, C17:1, C18:1c, C20:1, and C18:2n-6, Figure 6D), the explanation may be simple (only C22:0 suffered significant (*p* = 0.042) increase regarding the fresh fruits in both cultivars, in agreement with Figure 1B). This evolution causes the decrease of *ilr*9 after lye treatment. However, the C22:0 increase might be spurious and driven by the estimation procedure (the formation of an FA during processing is unrealistic). Particular explanations for other effects might also be possible.

**Figure 7:**
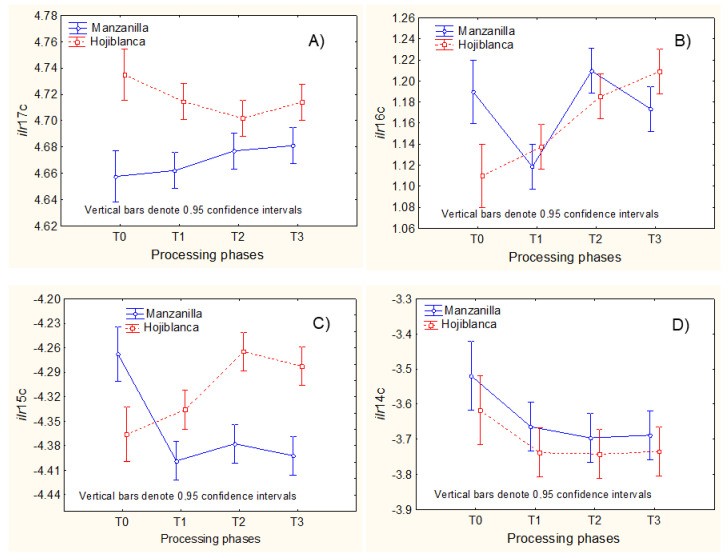
Examples of the influence of processing phases on *ilr coordinates* deduced from SBP following Ward’s clustering (“c” after the *ilr coordinate* number). (**A**–**D**) show *ilr coordinates* which showed significant differences between cultivars. T0, fresh olives; T1, lye-treated olives; T2, fermented olives; T3, packaged olives.

Regarding the *ilr coordinates* from Ward’s clustering (Figure 7), indicated by a “c” after the *ilr* number, *ilr*17c (C18:1c over the geometric mean of C17:1 and C17:0) only suffered slight non-significant changes (opposed for each cultivar) during processing (Figure 7A). However, in *ilr*14c (C15:0 over the geometric mean of C14:0, C17:0, C17:1, C18:1c, and C18:2n-6), the diminution after lye treatment in both cultivars (Figure 7D) could be caused by the partial decrease (possibly degradation) of C15:0 (mainly in Manzanilla), in agreement with Figure 1A. Still, the assignation is not conclusive due to the several FAs involved. Similar strategies can be applied to the remaining *ilr coordinates* from Ward’s clustering.

As observed, associating the effects on *ilr coordinates* to the involved FAs is not always straightforward in the current CoDA state of the art. Therefore, identifying the FAs responsible for the changes is sometimes complex and planning processing modifications to prevent their occurrence may still not be possible. The problem is currently under study, but the solutions are not simple [39].

No classification of Manzanilla and Hojiblanca table olive fat is intended in this work, which it is exclusively devoted to CoDA, since the “official” classification in the current legislation of olive oil is based on limits established in percentages [3]. However, this circumstance may be an opportunity for CoDA to be used to develop new standards defined according to the true structure of FA profiles. 

Furthermore, many ratios between FAs in oil chemistry/biochemistry are transcendent. As an example, the ratio of C18:1c/C16:0 is considered of interest in nutrition because palmitic acid has a regulatory influence on thrombogenic and fibrinolytic markers during the postprandial state in healthy subjects [40]; however, the recommended value (<5) [41] is deduced from percentages. Since CoDA is based on relative concentrations, using the new statistic may provide this and many other (log) ratios habitually found in the nutrition/olive oil literature in a natural way, with solid statistical support, and lead to easy handling and interpretable thresholds.

## 4. Conclusions

This work has demonstrated that green Manzanilla and Hojiblanca table olive FA profiles can be considered as compositional data and, consequently, statistically analysed with CoDA tools. In line with this hypothesis, the application of the typical CoDA exploratory tools was helpful to study the effects of green Spanish-style processing and packaging on the FA composition of the mentioned cultivars. A simple geometric mean barplot displayed the changes in each FA concerning the overall mean and allowed observation of the effects of the different elaboration steps and cultivar. Furthermore, CoDA exploratory techniques such as tetrahedral display, biplot, and CoDa dendrogram agree with the barplot and also displayed that the more relevant influences were observed among FA profiles of cultivars while the processing effects were scarce. 

The transformation of the original data in percentages into *ilr coordinates* allowed the application of standard multivariate techniques to the new data set. Canonical variate analysis confirmed the differences between cultivars and showed that Hojiblanca was more prone to suffer FA modifications than Manzanilla. LDA (bootstrapping = 1000) was 100% successful in assigning cultivars but the one-out cross-validation was not so efficient, with the *ilr coordinates* from Wards’ clustering leading to a greater level of successful LDA assignations than the decreasing order of variances (100 vs. 50% for MT1, MT3, or HT0). The MANOVA analysis found significant differences between cultivars and processing phases. The *p*-values were diverse between the same order of *ilr* balance (*coordinate*) due to the different basis; however, both showed that the first two *ilr coordinates* (based on the same FAs) were not significantly different between treatments (*p*-values: 1.663 (*ilr*1) and 0.609 (*ilr*2), decreasing order of variance; 0.176 (*ilr*1) and 0.742 (*ilr*2), based on Ward’s clustering). That is, their high variances were due to large errors because of the low proportion of the FA in the numerator. The GLM studied the MANOVA differences in detail (nested model), detecting significant differences in *ilr* balances (*coordinates*) between cultivars or changes during processing. The FAs responsible for the changes were identified in the simpler cases (i.e., the greater value of *ilr*16 in Manzanilla than in Hojiblanca could be assigned to higher values of C18:1C and C20:1, denominator in the formula, in Hojiblanca) but in most cases were hardly identifiable because of the complex *ilr* structures. 

Overall, according to the CoDA, table olive fatty acid profiles are different between cultivars and are scarcely affected by Spanish-style processing. Moreover, this work has demonstrated that fatty acid profiles, as compositional data, can be successfully studied by CoDA and that the same strategy can be applied to olive oils and fats in general, with the advantage of applying appropriate statistical techniques and preventing misinterpretations. This survey may represent a model for such statistical analyses. 

## Figures and Tables

**Table 1:** Correlation matrix (Pearson) among the complete fatty acid data set of Manzanilla and a sub-composition of it. To simplify, only the values of the whole data set corresponding to the fatty acids involved in the sub-composition are reproduced.

Complete data set (showing only the same fatty acids as the sub-composition)					
Variables	C16:0	C18:0	C16:1	C18:1c	C18:2n-6
C16:0	1	0.901	0.984	−0.990	0.284
C18:0	0.901	1	0.956	−0.883	−0.053
C16:1	0.984	0.956	1	−0.971	0.166
C18:1c	−0.990	−0.883	−0.971	1	−0.386
C18:2n-6	0.284	−0.053	0.166	−0.386	1
Sub-composition					
Variables	C16:0	C18:0	C16:1	C18:1c	C18:2n-6
C16:0	1	0.898	0.983	−0.994	0.254
C18:0	0.898	1	0.955	−0.892	−0.090
C16:1	0.983	0.955	1	−0.978	0.129
C18:1c	−0.994	−0.892	−0.978	1	−0.324
C18:2n-6	0.254	−0.090	0.129	−0.324	1

**Table 2:** Covariance matrix (Pearson) among the complete fatty acid data set of Manzanilla and a sub-composition of it. To simplify, only the values of the whole data set corresponding to the fatty acids involved in the sub-composition are reproduced.

Complete data set (showing only the same fatty acids as the sub-composition)					
Variables	C16:0	C18:0	C16:1	C18:1c	C18:2n-6
C16:0	0.741	0.247	0.226	−1.148	0.062
C18:0	0.247	0.101	0.081	−0.379	−0.004
C16:1	0.226	0.081	0.071	−0.349	0.011
C18:1c	−1.148	−0.379	−0.349	1.816	−0.132
C18:2n-6	0.062	−0.004	0.011	−0.132	0.064
Sub-composition					
Variables	C16:0	C18:0	C16:1	C18:1c	C18:2n-6
C16:0	0.738	0.248	0.229	−1.272	0.057
C18:0	0.248	0.104	0.083	−0.428	−0.008
C16:1	0.229	0.083	0.074	−0.395	0.009
C18:1c	−1.272	−0.428	−0.395	2.220	−0.125
C18:2n-6	0.057	−0.008	0.009	−0.125	0.067

**Table 3:** Overall (estimated from the complete set of fatty acid percentages) geometric means as well as geometric means of cultivar and processing phase treatments.

Fatty Acid	Overall	Manzanilla					Hojiblanca				
		Central Value	MT0	MT1	MT2	MT3	Central Value	HT0	HT1	HT2	HT3
C14:0	0.01727	0.01665	0.01895	0.01615	0.01656	0.01616	0.01792	0.01637	0.01723	0.01893	0.01848
C15:0	0.01469	0.01509	0.01825	0.01491	0.01449	0.01445	0.01430	0.01544	0.01388	0.01423	0.01425
C16:0	14.80477	15.63159	15.72515	15.36510	15.80978	15.67662	14.02169	13.70068	13.86287	14.22488	14.14256
C17:0	0.17269	0.17446	0.17526	0.17726	0.17339	0.17236	0.17094	0.16913	0.17065	0.17356	0.16955
C18:0	2.71359	3.03866	3.04994	3.09220	3.00853	3.01036	2.42330	2.43772	2.42016	2.42147	2.42109
C20:0	0.44878	0.47574	0.45768	0.48662	0.47267	0.47727	0.42336	0.41848	0.42589	0.41971	0.42696
C21:0	0.01521	0.01508	0.01442	0.01541	0.01516	0.01501	0.01534	0.01463	0.01528	0.01472	0.01645
C22:0	0.12186	0.12343	0.11186	0.12764	0.12160	0.12726	0.12031	0.11257	0.12018	0.12016	0.12467
C24:0	0.07529	0.07117	0.06304	0.07663	0.06924	0.07217	0.07966	0.07589	0.08112	0.07719	0.08270
C15:1	0.01226	0.00979	0.01108	0.00939	0.00975	0.00964	0.01536	0.01715	0.01752	0.01016	0.01929
C16:1	1.23830	1.52397	1.55456	1.49445	1.54750	1.51531	1.00618	0.94558	0.99666	1.03108	1.02253
C17:1	0.31149	0.31399	0.32053	0.32050	0.30803	0.31035	0.30902	0.30355	0.31189	0.31018	0.30774
C18:1c	72.65442	71.43034	71.14957	71.94538	71.04160	71.44762	73.89947	74.76980	74.25785	73.52064	73.49027
C20:1	0.28189	0.26268	0.25601	0.27037	0.25974	0.26145	0.30251	0.30635	0.30713	0.29807	0.30048
C18:2n-6	6.07551	6.08708	6.26793	5.81701	6.30417	6.06096	6.06396	5.64014	5.88087	6.21782	6.32298
C18:3n-3	0.90365	0.76257	0.76192	0.73946	0.77496	0.77418	1.07083	1.02414	1.06661	1.08145	1.08850
C18:3n-6	0.00460	0.00424	0.00558	0.00428	0.00370	0.00421	0.00500	0.00492	0.00403	0.00568	0.00549
C22:6n-3	0.01741	0.01743	0.01911	0.01424	0.02602	0.01364	0.01739	0.01674	0.01841	0.01994	0.01461
C18:2t	0.01139	0.01229	0.01229	0.01201	0.01277	0.01209	0.01057	0.00997	0.01033	0.01089	0.01079

Notes: M, Manzanilla; H, Hojiblanca; T0, fresh olives; T1, lye-treated olives; T2, fermented olives; T3, packaged olives. Treatments within cultivar are formed by combining cultivar and processing phases.

**Table 4:** Variation array matrix (upper half) and mean log-ratio (bottom half). The *clr* and total variances (*clr* variances sums) are shown in the last column on the right. In bold, the most relevant values.

Xi\Xj	C14:0	C15:0	C16:0	C17:0	C18:0	C20:0	C21:0	C22:0	C24:0	C15:1	C16:1	C17:1	C18:1c	C20:1	C18:2n-6	C18:3n-3	C18:3n-6	C22:6n-3	C18:2t	*clr* Variances	
C14:0		0.0123	0.0109	0.0054	0.0265	0.015	0.0094	0.0116	0.0157	**0.2405**	**0.0618**	0.005	0.0043	0.0069	0.0035	**0.0205**	**0.1304**	**0.0927**	0.0159	0.0043	
C15:0	−0.1622		0.0079	0.0075	0.0154	0.0125	0.0193	0.018	0.03	**0.257**	**0.0402**	0.0071	0.0094	0.0196	0.0095	**0.0475**	**0.1417**	**0.0943**	0.0115	0.0081	
C16:0	6.7535	6.9157		0.0025	0.0045	0.0023	0.01	0.0068	0.0227	**0.2793**	**0.0237**	0.0028	0.0056	0.0175	0.0036	**0.0506**	**0.1554**	**0.092**	0.0019	0.0049	
C17:0	2.3023	2.4645	−4.4512		0.0111	0.004	0.006	0.0047	0.0134	**0.2542**	**0.04**	0.0003	0.0011	0.0079	0.0024	**0.0336**	**0.1513**	**0.0919**	0.0067	0.0019	
C18:0	5.0568	5.219	−1.6967	2.7545		0.0036	0.0185	0.013	0.0349	**0.3145**	**0.0108**	0.0117	0.0172	0.0343	0.0153	**0.0813**	**0.1867**	**0.1037**	0.0047	0.0158	
C20:0	3.2573	3.4195	−3.4962	0.955	−1.7995		0.0065	0.0032	0.0164	**0.2822**	**0.0256**	0.0044	0.0066	0.0169	0.0071	**0.0538**	**0.1769**	**0.0948**	0.0049	0.0068	
C21:0	−0.1271	0.0351	−6.8806	−2.4294	−5.1839	−3.3845		0.0025	0.0049	**0.2458**	**0.0539**	0.006	0.0045	0.0068	0.0068	**0.0307**	**0.1709**	**0.0976**	0.0153	0.0057	
C22:0	1.9537	2.1159	−4.7998	−0.3486	−3.1032	−1.3037	2.0808		0.0065	**0.2653**	**0.0441**	0.0049	0.0045	0.0091	0.0063	**0.0373**	**0.1769**	**0.0975**	0.0112	0.0061	
C24:0	1.4722	1.6344	−5.2813	−0.8301	−3.5847	−1.7852	1.5993	−0.4815		**0.2353**	**0.0817**	0.0134	0.009	0.0048	0.0157	**0.0217**	**0.1864**	**0.1061**	0.0307	0.0127	
C15:1	−0.3425	−0.1803	−7.096	−2.6448	−5.3993	−3.5998	−0.2154	−2.2961	−1.8147		**0.3903**	**0.2542**	**0.2423**	**0.2251**	**0.2557**	**0.2025**	**0.3838**	**0.279**	**0.3004**	**0.2263**	
C16:1	4.2723	4.4345	−2.4812	1.97	−0.7845	1.015	4.3994	2.3186	2.8001	4.6148		**0.0408**	**0.0517**	**0.0803**	**0.0428**	**0.1431**	**0.2177**	**0.1316**	**0.0185**	**0.0469**	
C17:1	2.8922	3.0544	−3.8613	0.5899	−2.1647	−0.3652	3.0193	0.9385	1.42	3.2347	−1.3801		0.001	0.0075	0.0022	**0.0328**	**0.1462**	**0.0934**	0.0067	0.0017	
C18:1c	8.3443	8.5065	1.5908	6.042	3.2874	5.0869	8.4714	6.3906	6.8721	8.6868	4.072	5.4521		0.0034	0.0026	**0.0241**	**0.148**	**0.091**	0.0113	0.0016	
C20:1	2.7923	2.9545	−3.9612	0.49	−2.2645	−0.465	2.9194	0.8387	1.3202	3.1348	−1.48	−0.0999	−5.5519		0.009	**0.0115**	**0.1524**	**0.0967**	0.0256	0.0067	
C18:2n-6	5.8628	6.025	−0.8907	3.5605	0.806	2.6055	5.9899	3.9092	4.3906	6.2053	1.5905	2.9706	−2.4814	3.0705		**0.0303**	**0.1426**	**0.086**	0.0066	0.0021	
C18:3n-3	3.9572	4.1194	−2.7963	1.6549	−1.0996	0.6999	4.0844	2.0036	2.4851	4.2997	−0.3151	1.0651	−4.387	1.1649	−1.9056		**0.1485**	**0.1164**	**0.0622**	**0.0285**	
C18:3n-6	−1.3224	−1.1602	−8.0759	−3.6247	−6.3792	−4.5797	−1.1953	−3.2761	−2.7946	−0.9799	−5.5947	−4.2146	−9.6667	−4.1147	−7.1852	−5.2796		**0.2613**	**0.1556**	**0.1382**	
C22:6n-3	0.0078	0.17	−6.7457	−2.2945	−5.049	−3.2495	0.135	−1.9458	−1.4643	0.3503	−4.2645	−2.8843	−8.3364	−2.7845	−5.855	−3.9494	1.3302		**0.0918**	**0.0795**	
C18:2t	−0.4162	−0.254	−7.1697	−2.7185	−5.473	−3.6735	−0.289	−2.3698	−1.8883	−0.0737	−4.6885	−3.3083	−8.7604	−3.2085	−6.279	−4.3734	0.9062	−0.424		0.0092	
	Mean ln(Xi/Xj)																			0.6072	Total variance

**Table 5:** One-way ANOVA for testing significant differences among treatments, based on *ilr coordinates* obtained following *clr* variance and Ward’s clustering orders.

*ilr coordinate*	*ilr* Based on Decreasing *clr* Variance			*ilr* Based on Ward’s Clustering		
	Lambda de Wilks	F	Sig. (*p*-Value)	Lambda de Wilks	F	Sig. (*p*-Value)
*ilr*1	0.632	1.663	0.176	0.632	1.663	0.176
*ilr*2	0.824	0.609	0.742	0.824	0.609	0.742
*ilr*3	0.484	3.043	0.024	0.484	3.043	0.024
*ilr*4	0.019	149.806	0.000	0.013	209.621	0.000
*ilr*5	0.009	325.782	0.000	0.029	94.149	0.000
*ilr*6	0.010	273.113	0.000	0.586	2.023	0.103
*ilr*7	0.405	4.192	0.005	0.602	1.886	0.126
*ilr*8	0.217	10.307	0.000	0.099	26.110	0.000
*ilr*9	0.502	2.829	0.032	0.013	208.986	0.000
*ilr*10	0.105	24.386	0.000	0.535	2.484	0.052
*ilr*11	0.714	1.145	0.376	0.044	62.593	0.000
*ilr*12	0.064	42.078	0.000	0.464	3.295	0.017
*ilr*13	0.166	14.398	0.000	0.620	1.752	0.153
*ilr*14	0.440	3.630	0.011	0.471	3.207	0.019
*ilr*15	0.466	3.269	0.018	0.116	21.816	0.000
*ilr*16	0.169	14.088	0.000	0.185	12.555	0.000
*ilr*17	0.169	14.083	0.000	0.185	12.590	0.000
*ilr*18	0.118	21.269	0.000	0.720	1.113	0.393

Notes: gl1, 7, gl2, 20.

## Data Availability

Data is contained within the article or Supplementary Material.

## Supplementary Materials

The following supporting information can be downloaded at: https://www.mdpi.com/article/10.3390/foods11244024/s1, Figure S1. Tetrahedral display of the processing phases, according to cultivars, as a function of the fatty acid showing the largest *clr* variances. M, Manzanilla; H, Hojiblanca; T0, fresh olives; T1, lye-treated olives; T2, fermented olives; T3, packaged olives; Figure S2: CoDa form biplot, which preserves distances between treatments, helpful for studying differences among groups. PC1 accounted for 45.70% variance and PC2 for 26.07% (together, 71.77%). M, Manzanilla; H, Hojiblanca; T0, fresh olives; T1, lye-treated olives; T2, fermented olives; T3, packaged olives; Figure S3: Balance dendrogram, based on the sequential binary partition of FAs according to the descending order of variance. M, Manzanilla; H, Hojiblanca; T0, fresh olives; T1, lye-treated olives; T2, fermented olives; T3, packaged olives. Only first relevant balances are enumerated; Figure S4: Segregation of processing phases, according to cultivars, by canonical variate plot of *ilr* *coordinates*. The *coordinates* were obtained by following the *clr* decreasing variance for the first 11 fatty acids and just the variable order for the remaining balances. The overall centres for Manzanilla and Hojiblanca are also plotted. M, Manzanilla; H, Hojiblanca; T0, fresh olives; T1, lye-treated olives; T2, fermented olives; T3, packaged olives; Figure S5: Processing phase effects, according to cultivar, on *ilr coordinates*. Similar changes were also observed for *ilr*8, *ilr*6, *ilr*11c, *ilr*9c, *ilr*8c, *ilr*5c, and *ilr*4c (with “c” indicating obtained following Ward’s clustering), but with the values of Manzanilla always above those of Hojiblanca. T0, fresh olives; T1, lye-treated olives; T2, fermented olives; T3, packaged olives; Table S1: Fatty acid composition of the Manzanilla and Hojiblanca fat throughout the Spanish-style processing and packaging; Table S2. Sequential binary partition (SBP) matrix using the decreasing order of fatty acid *clr* variances (except last five balances, which just followed the variable order of the remaining acids); Table S3. *Ilr coordinates* obtained by SBP based on the decreasing order of the fatty acid *clr* variance (except last five balances, which just followed the variable order of the remaining acids); Table S4. SBP matrix, obtained following Ward’s clustering sequence; Table S5. *Ilr coordinates* obtained by SBP, based on Ward’s clustering sequence; Table S6. Means and variances of the balances obtained by SBP based on the decreasing *clr* variance and Ward’s clustering sequence; Table S7. Proportion of successful assignation after application of Linear Discriminant Analysis, using bootstrapping (1000), to the *ilr coordinates* based on the *clr* variance decreasing order and Ward’s clustering sequences.

## Appendix A

Succinct information on the fundamental compositional data analysis statistics used in the manuscript.

**1. Central tendency and dispersion**

The standard means and standard deviation (developed for the Euclidean space) have no meaning in CoDa and are substituted by the following concepts [23].

(a) Central tendency. The best representation of the centre of a dataset (X) of size n and D parts is the closed geometric mean (barycenter):
  $$g_{m}=C\left[g_{1},\;g_{2},\ldots .g_{D}\right]\;\mathrm{where}\;g_{j}={\left(\prod_{i=1}^{n}x_{ij}\right)}^{1/n}$$
  where the *C* operator stands for closure, which rescales *g_j_* to a constant (1 or 100). The dispersion is indicated by their appropriate 0, 25, 50 (median), 75, and 100 percentiles, although they are rarely used in CoDa analysis (CoDA).
(b) Variability.

The variability (of X) is evaluated through the matrix of log-ratio variances (variance array) since, by definition, these data always carry relative information:

$$T=\left(\begin{array}{cccc} t_{11} & t_{12} & \ldots & t_{1D}\\ t_{21} & t_{22} & \ldots & t_{2D}\\ \ldots & \ldots & \ldots & \ldots \\ t_{D1} & t_{D2} & \ldots & t_{DD} \end{array}\right)\;\mathrm{where}\;t_{ij}=var\left(ln\frac{x_{i}}{x_{j}}\right)$$

where diagonal terms are zeros (log-ratio of each component by itself) and is symmetric with respect to the diagonal. To prevent unnecessary repetition, the bottom half array is used to show the corresponding (average over treatment) log-ratios $\left(ln\frac{x_{i}}{xj}\right)$. The total variance of components is summarized by:

$$totvar\left(X\right)=\frac{1}{2D}\overset{D}{\underset{i=1}{\sum }}\overset{D}{\underset{j=1}{\sum }}var\left(ln\frac{x_{i}}{xj}\right)$$

**2. Log-ratio transformation of compositional data**

The most common transformations are the centred log-ratio (*clr*) and the isometric log-ratio (*ilr*), which leads to orthonormal *coordinates* in the Euclidean space.

(a) Centred log-ratio transformation *(clr)*.

It is defined as:

$$Z=clr\left(X\right)=\left[ln\frac{x_{1}}{g_{m}\left(x\right)},\;\ldots ,ln\frac{x_{D}}{g_{m}\left(x\right)}\right]$$

which is estimated row-wise and, apart from CoDa biplot, has diverse applications since it allows relating results directly to the original variables. The variance of each *clr* part (*clr* variance) is estimated column-wise by the standard method [6].

(b) Isometric log-ratio transformation (*ilr*).

They are obtained by the successive partition of the composition into two mutually exclusive groups (SBP). The process ends when the group has only two single parts. The number of transformed values is always (D parts-1). Their normalized log-ratios (balances or coordinates) are estimated, row-wise, by:

$$z_{i}=\sqrt{\frac{rs}{r+s}}\;ln\frac{{\left(x_{i1}x_{i2\ldots }x_{ir}\right)}^{1/r}}{{\left(x_{i1}x_{i2\ldots }x_{is}\right)}^{1/s}}$$

The parts in the numerator, denominator, or absent are coded by 1, −1, and 0, respectively, and are gathered in a matrix, which is required for back-transformation of the obtained *coordinates* into the original units [6].

Readers are encouraged to consult specialised texts for more detailed information on CoDA techniques [6,7,17,33]. Nevertheless, most of the techniques are briefly commented on when introduced in the text. The standard multivariate techniques were always applied to log-ratios, *ilr*-transformed data (usually known as *coordinates*).
