# Supplementary material for: Effect of Spanish-Style Table Olive Processing on Fatty Acid Profile: A Compositional Data Analysis (CoDA) Approach
Source: Foods. 2022 Dec 13;11(24):4024. doi: 10.3390/foods11244024 (PMC9777950; doi:10.3390/foods11244024)
Supplement: Supplementary file 1 [file foods-11-04024-s001.zip › foods-2042774-supplementary.pdf]

Supplementary Information to

Effect of Spanish-style table olive processing on fatty acid profile: a compositional data analysis (CoDA) approach

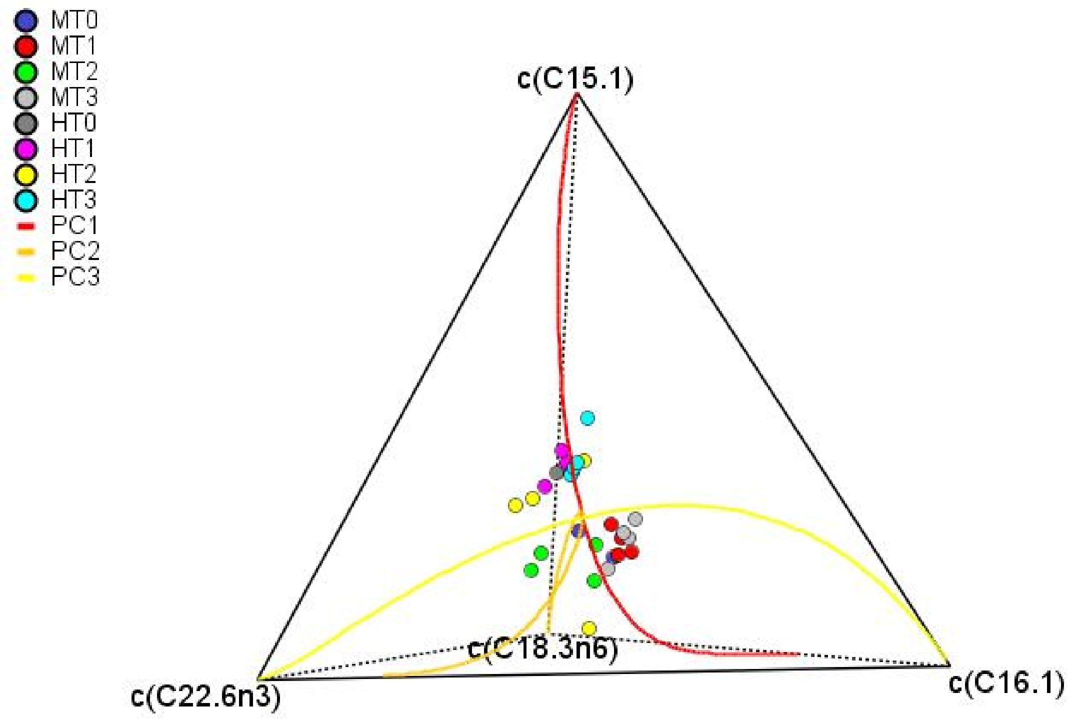

Figure S1. Tetrahedral display of the processing phases, according to cultivars, as a function of the fatty acid showing the largest *clr* variances. M, Manzanilla; H, Hojiblanca; T0, fresh olives; T1, lye-treated olives; T2, fermented olives; T3, packaged olives.

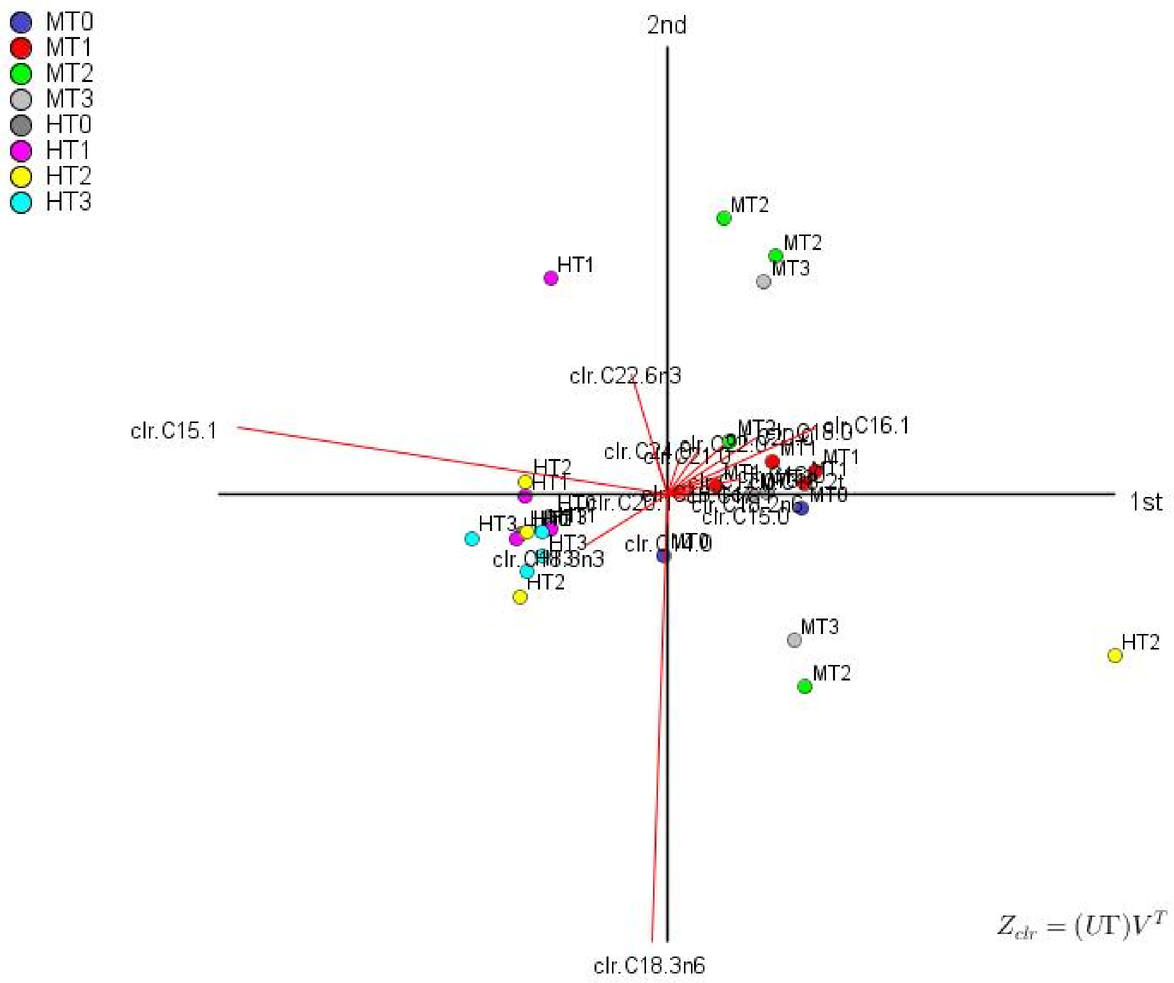

Figure S2. CoDa form biplot, which preserves distances between treatments, helpful for studying differences among groups. PC1 accounted for 45.70% variance and PC2 for 26.07% (together, 71.77%). M, Manzanilla; H, Hojiblanca; T0, fresh olives; T1, lye-treated olives; T2, fermented olives; T3, packaged olives.

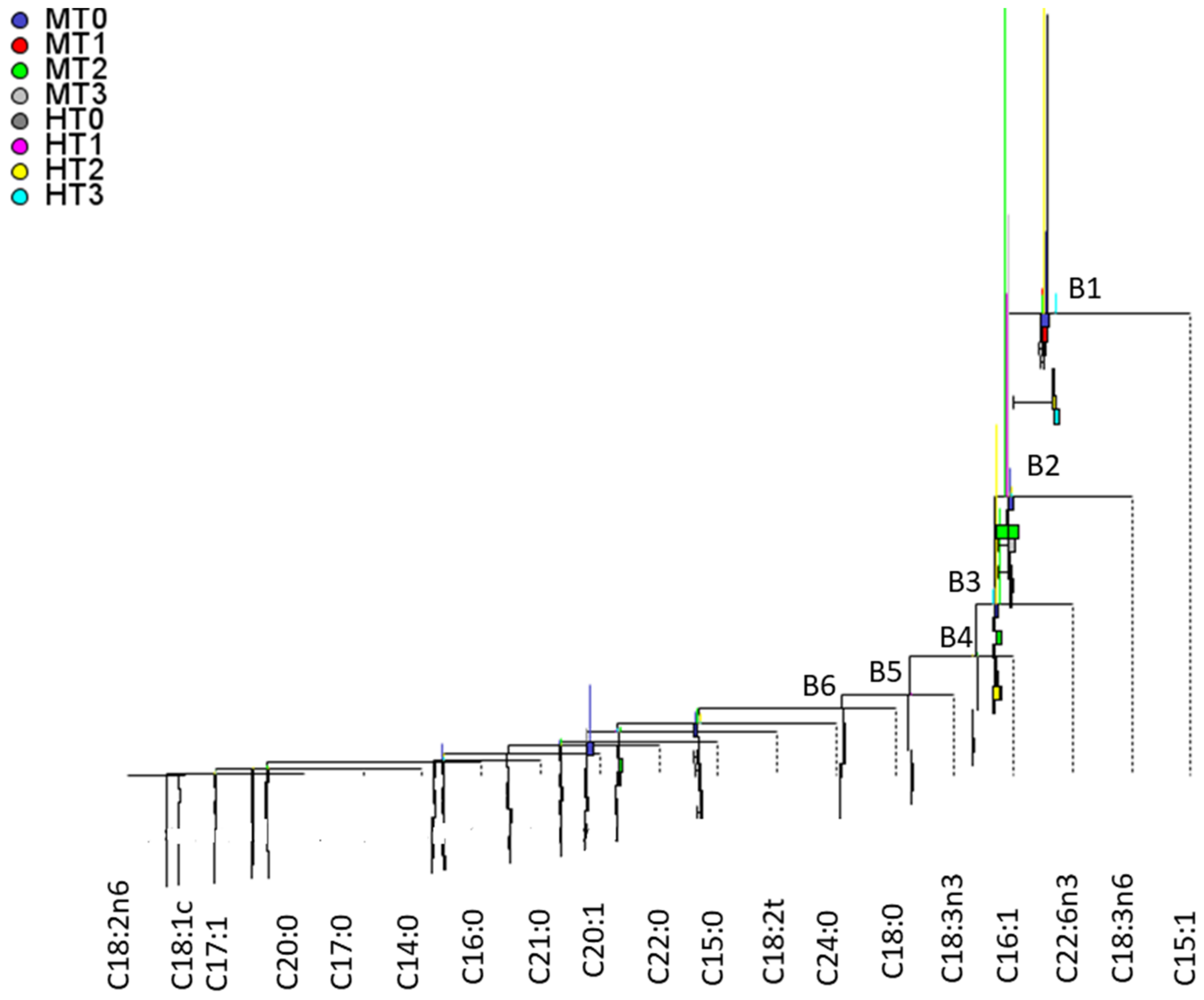

Figure S3. Balance dendrogram, based on the sequential binary partition of FAs according to the descending order of variance. M, Manzanilla; H, Hojiblanca; T0, fresh olives; T1, lye-treated olives; T2, fermented olives; T3, packaged olives. Only first relevant balances are enumerated.

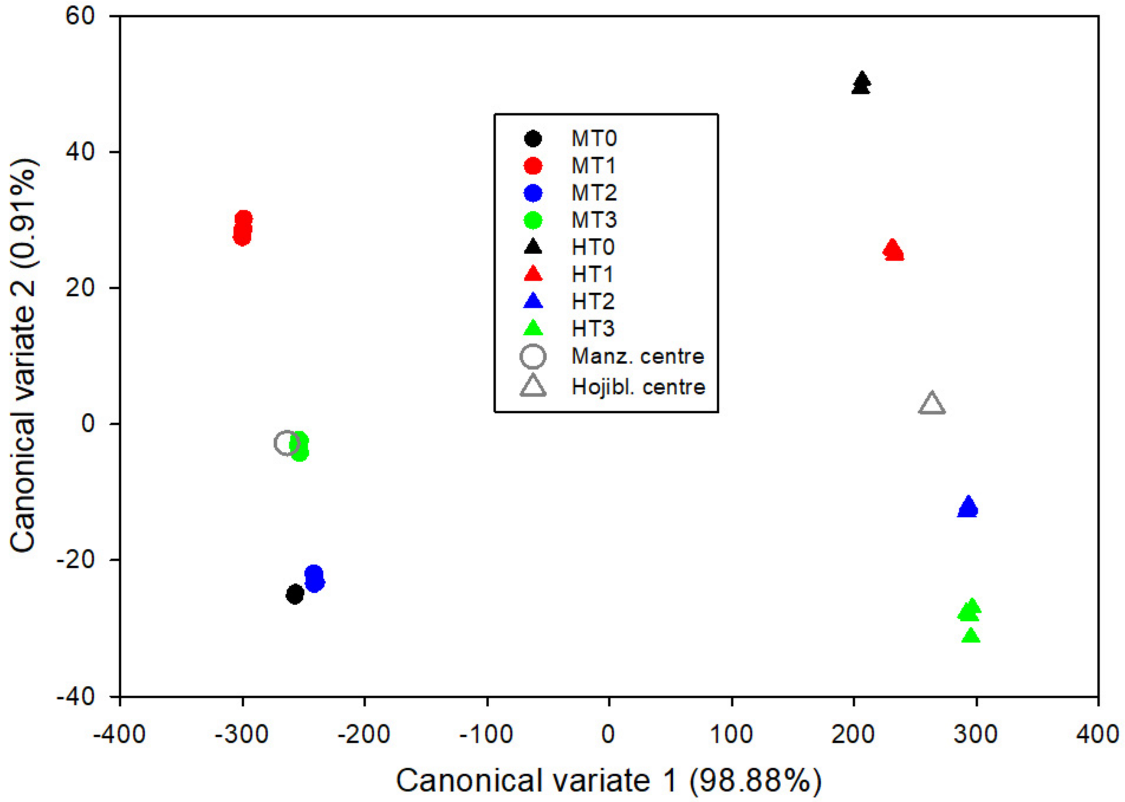

Figure S4. Segregation of processing phases, according to cultivars, by canonical variate plot of *ilr coordinates*. The *coordinates* were obtained by following the *clr* decreasing variance for the first 11 fatty acids and just the variable order for the remaining balances. The overall centres for Manzanilla and Hojiblanca are also plotted. M, Manzanilla; H, Hojiblanca; T0, fresh olives; T1, lye-treated olives; T2, fermented olives; T3, packaged olives.

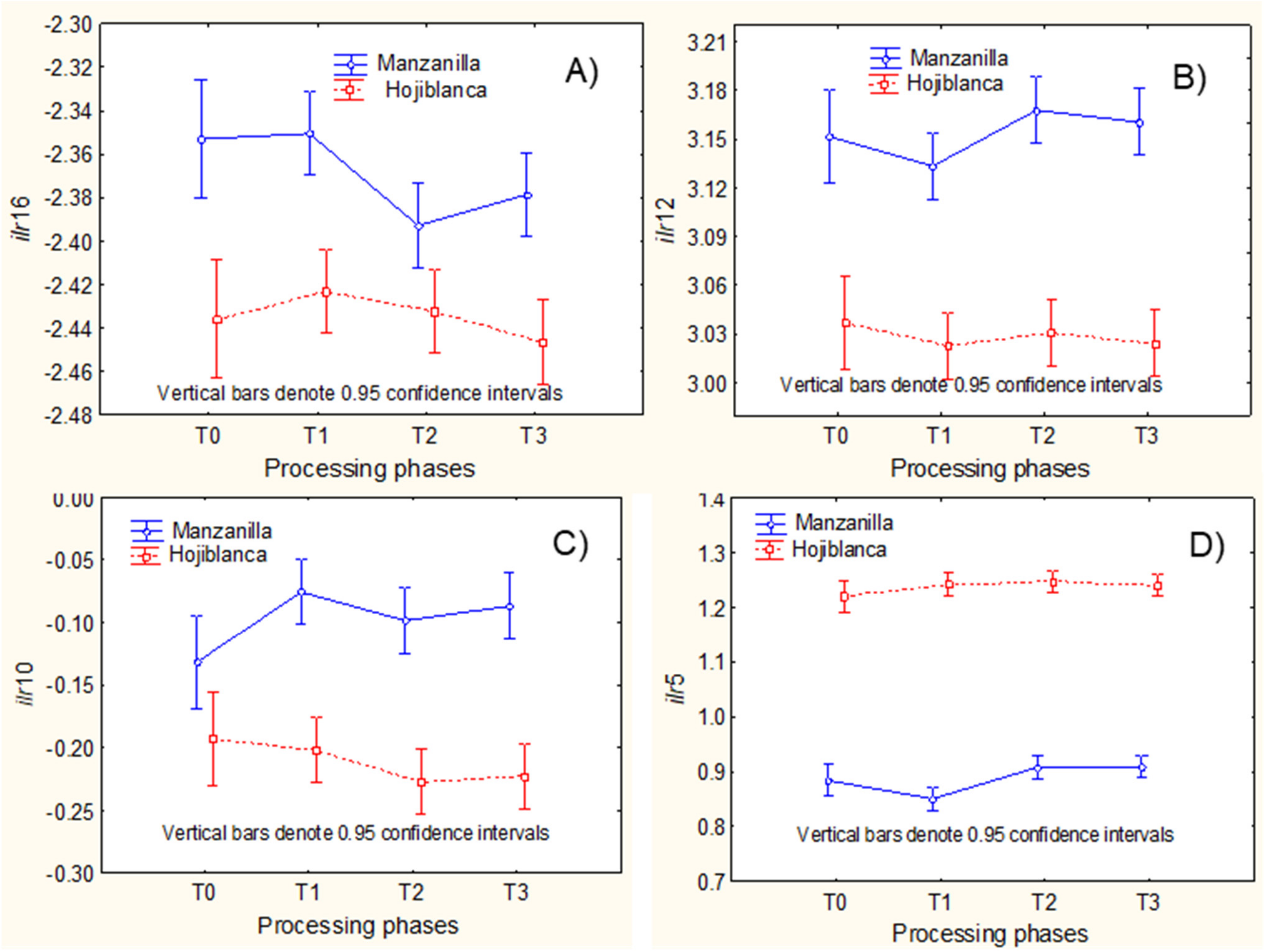

Figure S5. Examples of processing phase effects, according to cultivar, on *ilr coordinates*. Similar changes were also observed for *ilr*8, *ilr*6, *ilr*11c, *ilr*9c, *ilr*8c, *ilr*5c, and *ilr*4c (with "c" indicating obtained following Wards' clustering), but with the values of Manzanilla always above those of Hojiblanca. T0, fresh olives; T1, lye-treated olives; T2, fermented olives; T3, packaged olives.

Table S1. Fatty acid composition of the Manzanilla and Hojiblanca fat throughout the Spanish-style processing and packaging

Processing phases	C14:0	C15:0	C16:0	C17:0	C18:0	C20:0	C21:0	C22:0	C24:0
MT0	0.01819144	0.02209452	16.01647673	0.17695961	3.00660896	0.43548211	0.01267640	0.10201372	0.05658797
MT0	0.01974274	0.01506705	15.43911768	0.17358366	3.09388960	0.48101600	0.01639576	0.12266403	0.07022228
MT1	0.01619250	0.01489129	15.32751279	0.17852700	3.11259005	0.48789519	0.01638129	0.12704793	0.07600453
MT1	0.01585988	0.01466476	15.30461700	0.17882003	3.11535761	0.48887806	0.01510545	0.12807416	0.07671038
MT1	0.01600493	0.01493883	15.42929098	0.17628149	3.07335189	0.48438784	0.01535012	0.12719250	0.07688859
MT1	0.01656373	0.01515627	15.39931934	0.17542700	3.06782226	0.48531346	0.01485393	0.12824578	0.07693991
MT2	0.01783843	0.01495339	16.32480598	0.17603312	2.91692152	0.43770889	0.01370964	0.10525300	0.05686604
MT2	0.01588252	0.01425845	15.75249715	0.17202384	2.97182230	0.47495950	0.01536733	0.12430759	0.07102730
MT2	0.01626946	0.01451874	15.57961718	0.17239412	3.07195307	0.49078039	0.01608591	0.12843788	0.07513976
MT2	0.01632230	0.01424048	15.59370739	0.17313595	3.07650726	0.48919941	0.01559145	0.13010001	0.07575141
MT3	0.01639771	0.01489417	15.97066591	0.17023197	2.90699662	0.44870701	0.01370849	0.12135317	0.06221545
MT3	0.01601298	0.01417752	15.66401939	0.16965921	2.94727694	0.47323234	0.01501822	0.12496549	0.07328204
MT3	0.01614799	0.01446722	15.53147926	0.17516543	3.09502750	0.49464162	0.01571876	0.13156045	0.07654705
MT3	0.01609130	0.01427646	15.54428518	0.17444361	3.09699878	0.49399876	0.01569223	0.13145270	0.07772950
HT0	0.01642946	0.01380264	13.79311972	0.17002803	2.43051051	0.41312745	0.01443968	0.11004751	0.07279613
HT0	0.01630365	0.01726366	13.60886868	0.16823550	2.44494839	0.42390310	0.01482830	0.11516034	0.07910780
HT1	0.01734899	0.01370005	13.85951062	0.16581254	2.37600813	0.42349706	0.01530611	0.11867201	0.07877640
HT1	0.01696225	0.01383817	13.86741605	0.16603726	2.38205952	0.42439684	0.01525481	0.12003604	0.08210259
HT1	0.01748388	0.01384537	13.90093755	0.17574627	2.46584931	0.42715806	0.01517200	0.12125931	0.08057018
HT1	0.01712058	0.01413787	13.82372603	0.17526001	2.45815015	0.42854218	0.01538933	0.12075564	0.08308797
HT2	0.01943200	0.01466911	14.56193106	0.18035091	2.37407252	0.39436672	0.01313220	0.10979514	0.06547840
HT2	0.01844668	0.01400056	14.22626228	0.17014377	2.42322332	0.42284613	0.01477025	0.12204910	0.07808236
HT2	0.01878149	0.01398621	14.01499271	0.17146501	2.43643649	0.43194016	0.01563140	0.12373334	0.08193625
HT2	0.01906431	0.01427348	14.10235143	0.17247427	2.45287153	0.43081222	0.01549079	0.12572543	0.08474129
HT3	0.01833454	0.01444710	14.18297304	0.16913007	2.41750176	0.42735741	0.01594227	0.12695875	0.08224565
HT3	0.01862508	0.01429541	14.17116046	0.16868738	2.41861545	0.42755185	0.01580309	0.12640107	0.08133621
HT3	0.01883991	0.01420513	14.13163301	0.17049325	2.42252325	0.42370003	0.01771585	0.12182217	0.08420568
HT3	0.01814159	0.01404182	14.08469511	0.16991148	2.42571424	0.42923088	0.01639517	0.12356878	0.08305269

Notes: nd, below detection limits. M, Manzanilla; H, Hojiblanca; T0, fresh olives; T1, lye-treated olives; T2, fermented olives; T3, packaged olives.

Table S1. Fatty acid composition of the Manzanilla and Hojiblanca fat throughout the Spanish-style processing and packaging (cont)

Processing phases	C15:1	C16:1	C17:1	C18:1c	C20:1	C18:2n-6	C18:3n-3	C18:3n-6	C22:6n-3	C18:2t
MT0	0.01452356	1.59322581	0.32193622	70.90888085	0.24479435	6.26846363	0.75859427	0.00651657	0.02352943	0.01244386
MT0	0.00844596	1.51683032	0.31912998	71.39108140	0.26773382	6.26738650	0.76525210	0.00478502	0.01551556	0.01214054
MT1	0.00963998	1.47903866	0.32236285	72.04829657	0.27086054	5.74217606	0.74009442	0.00407499	0.01400542	0.01240795
MT1	0.00831314	1.48084628	0.32074704	72.06407555	0.27063107	5.74630629	0.74043033	0.00435895	0.01459052	0.01161349
MT1	0.00809581	1.50755050	0.31954656	71.83862574	0.27005440	5.87651410	0.73722780	0.00401273	0.01297982	0.01170538
MT1	0.01199730	1.51066977	0.31935294	71.83084412	0.26993802	5.90492253	0.74007957	0.00471239	0.01550553	0.01233614
MT2	0.00817240	1.62518848	0.31904725	70.49781776	0.24686436	6.41993293	0.77688898	0.00957517	0.01861081	0.01381186
MT2	0.01119185	1.55096717	0.30223893	70.97773462	0.26453205	6.45522172	0.77766455	nd	0.03363444	0.01266873
MT2	0.01051373	1.50661669	0.30281333	71.35234251	0.26433044	6.17292480	0.77215211	0.00487095	0.03578151	0.01245741
MT2	0.00939030	1.51013808	0.30832532	71.34192777	0.26367708	6.17417813	0.77314538	nd	0.02047263	0.01218968
MT3	0.00859240	1.57235902	0.31893097	71.03366081	0.25195464	6.28264996	0.77078332	0.00780531	0.01526249	0.01283058
MT3	0.00977153	1.53497460	0.30931720	71.28642969	0.26446061	6.29254988	0.77479834	0.00440599	0.01299789	0.01265016
MT3	0.01027435	1.47752710	0.30526650	71.74057390	0.26509986	5.84505561	0.77625995	0.00455433	0.01306207	0.01157107
MT3	0.01001015	1.47850436	0.30804371	71.73236796	0.26453971	5.83993662	0.77488632	nd	0.01335478	0.01138791
HT0	0.01776647	0.95139093	0.30226200	74.69590650	0.30118060	5.64056323	1.02310172	0.00521040	0.01854508	0.00977195
HT0	0.01654884	0.93980148	0.30484867	74.84376158	0.31161034	5.63971902	1.02517639	0.00463800	0.01510674	0.01016952
HT1	0.01799318	0.98879517	0.30677526	74.27240592	0.31075953	5.89956437	1.09690865	0.00570946	0.02256427	0.00989228
HT1	0.01794268	0.98614509	0.30577743	74.28842376	0.31127780	5.88032048	1.09050674	0.00452410	0.01705780	0.00992061
HT1	0.01720732	1.00757879	0.31834403	74.18888966	0.30246334	5.87572873	1.04038159	nd	0.01877594	0.01060873
HT1	0.01694465	1.00431253	0.31688660	74.28172344	0.30413613	5.86790768	1.03998906	0.00509779	0.01589711	0.01093526
HT2	0.01940664	1.06509295	0.31142944	73.18144516	0.28399334	6.29312887	1.08170476	0.00612227	0.01377131	0.01067720
HT2	0.00173287	1.03225314	0.30749201	73.50817291	0.30069813	6.25685677	1.07320564	0.00557270	0.01311477	0.01107661
HT2	0.01783348	1.01454030	0.31431993	73.71923490	0.30377288	6.18557893	1.09205113	0.00593883	0.02691815	0.01090840
HT2	0.01775209	1.01326827	0.30755029	73.67490641	0.30427800	6.13691808	1.07893699	0.00515397	0.03252487	0.01090627
HT3	0.01753836	1.02972838	0.30647279	73.38769578	0.30099672	6.37636466	1.09348250	0.00571156	0.01636091	0.01075776
HT3	0.01769362	1.02559478	0.30720126	73.42175672	0.30144284	6.36160590	1.09033458	0.00514272	0.01579125	0.01096034
HT3	0.02369237	1.02025623	0.30735173	73.56472751	0.29916270	6.27184895	1.08025500	0.00518097	0.01222615	0.01016009
HT3	0.01882293	1.01458986	0.30995375	73.58712137	0.30031713	6.28276196	1.08997947	0.00595191	0.01442556	0.01132430

Notes: nd. below detection limits. M. Manzanilla; H. Hojiblanca; T0. fresh olives; T1. lye-treated olives; T2. fermented olives; T3. packaged olives.

Table S2. Sequential binary partition (SBP) matrix using the decreasing order of fatty acid *clr* variances (except the last five balances. which just followed the variable order of the remaining acids).

Balance	C14:0	C15:0	C16:0	C17:0	C18:0	C20:0	C21:0	C22:0	C24:0	C15:1	C16:1	C17:1	C18:1c	C20:1	C18:2n-6	C18:3n-3	C18:3n-6	C22:6n-3	C18:2t
*ilr*1	-1	-1	-1	-1	-1	-1	-1	-1	-1	1	-1	-1	-1	-1	-1	-1	-1	-1	-1
*ilr*2	-1	-1	-1	-1	-1	-1	-1	-1	-1	0	-1	-1	-1	-1	-1	-1	1	-1	-1
*ilr*3	-1	-1	-1	-1	-1	-1	-1	-1	-1	0	-1	-1	-1	-1	-1	-1	0	1	-1
*ilr*4	-1	-1	-1	-1	-1	-1	-1	-1	-1	0	1	-1	-1	-1	-1	-1	0	0	-1
*ilr*5	-1	-1	-1	-1	-1	-1	-1	-1	-1	0	0	-1	-1	-1	-1	1	0	0	-1
*ilr*6	-1	-1	-1	-1	1	-1	-1	-1	-1	0	0	-1	-1	-1	-1	0	0	0	-1
*ilr*7	-1	-1	-1	-1	0	-1	-1	-1	1	0	0	-1	-1	-1	-1	0	0	0	-1
*ilr*8	-1	-1	-1	-1	0	-1	-1	-1	0	0	0	-1	-1	-1	-1	0	0	0	1
*ilr*9	-1	1	-1	-1	0	-1	-1	-1	0	0	0	-1	-1	-1	-1	0	0	0	0
*ilr*10	-1	0	-1	-1	0	1	-1	-1	0	0	0	-1	-1	-1	-1	0	0	0	0
*ilr*11	-1	0	-1	-1	0	0	1	-1	0	0	0	-1	-1	-1	-1	0	0	0	0
*ilr*12	-1	0	1	-1	0	0	0	-1	0	0	0	-1	-1	-1	-1	0	0	0	0
*ilr*13	1	0	0	-1	0	0	0	-1	0	0	0	-1	-1	-1	-1	0	0	0	0
*ilr*14	0	0	0	1	0	0	0	-1	0	0	0	-1	-1	-1	-1	0	0	0	0
*ilr*15	0	0	0	0	0	0	0	1	0	0	0	-1	-1	-1	-1	0	0	0	0
*ilr*16	0	0	0	0	0	0	0	0	0	0	0	1	-1	-1	-1	0	0	0	0
*ilr*17	0	0	0	0	0	0	0	0	0	0	0	0	1	-1	-1	0	0	0	0
*ilr*18	0	0	0	0	0	0	0	0	0	0	0	0	0	1	-1	0	0	0	0

Table S3. *Ilr coordinates* obtained by SBP based on the decreasing order of the fatty acid *clr* variance (except last five balances. which just followed the variable order of the remaining acids)

Processing phases	*ilr*1	*ilr*2	*ilr*3	*ilr*4	*ilr*5	*ilr*6	*ilr*7	*ilr*8	*ilr*9
MT0	-2.74270	-3.72421	-2.62670	1.55717	0.89659	2.39218	-1.55114	-3.26819	-2.97799
MT0	-3.25549	-4.02634	-3.05802	1.47731	0.87113	2.38546	-1.36351	-3.31545	-3.40539
MT1	-3.10450	-4.16806	-3.14832	1.46088	0.84508	2.39841	-1.27342	-3.27741	-3.39887
MT1	-3.24550	-4.09542	-3.09853	1.47287	0.85710	2.41178	-1.25029	-3.33102	-3.40428
MT1	-3.26291	-4.17173	-3.21474	1.48843	0.85075	2.39537	-1.25147	-3.32650	-3.38818
MT1	-2.90271	-4.03029	-3.04711	1.48539	0.84935	2.38799	-1.25687	-3.27825	-3.37521
MT2	-3.30285	-3.32874	-2.84563	1.58681	0.93239	2.37447	-1.53365	-3.14535	-3.36227
MT2	-2.95906	-4.90026	-2.28823	1.52072	0.91115	2.36998	-1.32646	-3.24260	-3.42811
MT2	-3.07908	-4.04687	-2.23684	1.48156	0.89195	2.39112	-1.27953	-3.26794	-3.41925
MT2	-3.10937	-4.87858	-2.77697	1.48561	0.89519	2.39481	-1.26865	-3.28727	-3.43794
MT3	-3.23237	-3.51608	-3.03814	1.55260	0.92185	2.36780	-1.44373	-3.21847	-3.36924
MT3	-3.08087	-4.07666	-3.20883	1.51190	0.90863	2.36249	-1.29339	-3.24081	-3.43058
MT3	-3.03853	-4.04946	-3.20904	1.46720	0.90228	2.40447	-1.25359	-3.33620	-3.42035
MT3	-3.01956	-4.84940	-3.18638	1.46911	0.90180	2.40646	-1.23614	-3.34992	-3.43256
HT0	-2.47091	-3.87444	-2.80084	1.08514	1.23528	2.22483	-1.24836	-3.45455	-3.42207
HT0	-2.54712	-4.00170	-3.02724	1.04325	1.20528	2.19665	-1.19841	-3.44545	-3.21926
HT1	-2.49831	-3.82233	-2.63766	1.09609	1.27918	2.17616	-1.19514	-3.46638	-3.45569
HT1	-2.47513	-4.03438	-2.91108	1.09118	1.27064	2.17571	-1.15524	-3.46320	-3.44469
HT1	-2.48599	-4.84276	-2.82815	1.10255	1.21183	2.19724	-1.18757	-3.40862	-3.45469
HT1	-2.54580	-3.92736	-2.99326	1.09532	1.20708	2.18928	-1.16089	-3.38010	-3.43331
HT2	-2.39847	-3.72276	-3.11298	1.17681	1.27408	2.18432	-1.37792	-3.39221	-3.38284
HT2	-4.76107	-3.83138	-3.18159	1.12187	1.23960	2.17674	-1.22421	-3.37062	-3.44663
HT2	-2.54311	-3.81999	-2.49391	1.09345	1.24516	2.17028	-1.18670	-3.39614	-3.45962
HT2	-2.55282	-3.97141	-2.31338	1.08893	1.22912	2.17257	-1.15619	-3.39834	-3.44050
HT3	-2.53199	-3.83121	-2.97882	1.10680	1.24540	2.16109	-1.18458	-3.41230	-3.42873
HT3	-2.51539	-3.93069	-3.01272	1.10316	1.24269	2.16165	-1.19604	-3.39366	-3.43895
HT3	-2.21822	-3.90931	-3.26144	1.09726	1.23217	2.16166	-1.16161	-3.47164	-3.45150
HT3	-2.45901	-3.78438	-3.10140	1.09103	1.24055	2.16273	-1.17618	-3.35976	-3.45485

Note: M. Manzanilla; H. Hojiblanca; T0. fresh olives; T1. lye-treated olives; T2. fermented olives; T3. packaged olives.

Table S3. *Ilr coordinates* obtained by SBP based on the decreasing order of the fatty acid *clr* variance (except last five balances. which just followed the variable order of the remaining acids) (cont)

Processing phases	*ilr*10	*ilr*11	*ilr*12	*ilr*13	*ilr*14	*ilr*15	*ilr*16	*ilr*17	*ilr*18
MT0	-0.14992	-3.91886	3.19116	-3.63886	-1.81346	-2.83685	-2.33532	3.30461	-2.29305
MT0	-0.11406	-3.71137	3.11214	-3.60206	-1.88066	-2.69152	-2.37067	3.27364	-2.22959
MT1	-0.07953	-3.68875	3.13097	-3.78660	-1.85109	-2.64744	-2.34267	3.31211	-2.15950
MT1	-0.06723	-3.76314	3.13171	-3.80655	-1.85016	-2.63914	-2.34705	3.31234	-2.16060
MT1	-0.07866	-3.74910	3.13912	-3.79692	-1.86440	-2.64831	-2.35525	3.30151	-2.17796
MT1	-0.07755	-3.78472	3.13178	-3.76623	-1.87101	-2.64176	-2.35701	3.29963	-2.18167
MT2	-0.15786	-3.85005	3.20580	-3.66371	-1.82714	-2.81280	-2.35077	3.28667	-2.30398
MT2	-0.09439	-3.74464	3.16514	-3.79756	-1.88353	-2.67007	-2.42114	3.26176	-2.25898
MT2	-0.06913	-3.70268	3.15206	-3.77474	-1.88054	-2.63227	-2.40789	3.28462	-2.22790
MT2	-0.07279	-3.73647	3.14810	-3.77680	-1.88182	-2.62426	-2.39157	3.28543	-2.22979
MT3	-0.13523	-3.85115	3.18119	-3.75940	-1.88482	-2.68683	-2.35293	3.29335	-2.27427
MT3	-0.09497	-3.76580	3.15969	-3.78893	-1.89744	-2.66573	-2.39490	3.27583	-2.24112
MT3	-0.05834	-3.72342	3.14993	-3.78196	-1.86340	-2.60224	-2.38755	3.31014	-2.18725
MT3	-0.05921	-3.72481	3.15104	-3.78537	-1.86811	-2.60431	-2.37881	3.31127	-2.18813
HT0	-0.20049	-3.78137	3.04806	-3.75271	-1.88034	-2.78933	-2.43432	3.30555	-2.07184
HT0	-0.18498	-3.76317	3.02592	-3.77205	-1.90641	-2.75864	-2.43730	3.29333	-2.04767
HT1	-0.20366	-3.74940	3.02714	-3.72323	-1.93261	-2.74093	-2.44185	3.26980	-2.08145
HT1	-0.19983	-3.75094	3.02960	-3.74536	-1.93261	-2.72967	-2.44427	3.27063	-2.07796
HT1	-0.20461	-3.76893	3.01760	-3.72910	-1.88431	-2.72271	-2.40048	3.28158	-2.09772
HT1	-0.19960	-3.75167	3.01601	-3.74760	-1.88623	-2.72662	-2.40603	3.28089	-2.09288
HT2	-0.27029	-3.91078	3.06077	-3.61533	-1.83708	-2.80483	-2.41718	3.26812	-2.19080
HT2	-0.21814	-3.80176	3.03379	-3.67750	-1.91746	-2.71983	-2.44431	3.25078	-2.14630
HT2	-0.20899	-3.75399	3.01138	-3.66779	-1.91720	-2.71284	-2.42575	3.25365	-2.13100
HT2	-0.21203	-3.76421	3.01610	-3.65290	-1.90953	-2.69215	-2.44263	3.25570	-2.12425
HT3	-0.22026	-3.73440	3.02529	-3.69061	-1.93284	-2.68790	-2.45246	3.24132	-2.15898
HT3	-0.21995	-3.74383	3.02308	-3.67528	-1.93480	-2.69228	-2.45030	3.24204	-2.15629
HT3	-0.23668	-3.63175	3.02503	-3.65763	-1.91480	-2.72095	-2.44414	3.25253	-2.15161
HT3	-0.21453	-3.70292	3.02360	-3.69647	-1.92314	-2.71142	-2.43854	3.25049	-2.15012

Note: M. Manzanilla; H. Hojiblanca; T0. fresh olives; T1. lye-treated olives; T2. fermented olives; T3. packaged olives.

Table S4. SBP matrix. obtained following Ward's clustering sequence

Balance	C14:0	C15:0	C16:0	C17:0	C18:0	C20:0	C21:0	C22:0	C24:0	C15:1	C16:1	C17:1	C18:1c	C20:1	C18:2n-6	C18:3n-3	C18:3n-6	C22:6n-3	C18:2t
*ilr*1c	-1	-1	-1	-1	-1	-1	-1	-1	-1	1	-1	-1	-1	-1	-1	-1	-1	-1	-1
*ilr*2c	-1	-1	-1	-1	-1	-1	-1	-1	-1	0	-1	-1	-1	-1	-1	-1	1	-1	-1
*ilr*3c	-1	-1	-1	-1	-1	-1	-1	-1	-1	0	-1	-1	-1	-1	-1	-1	0	1	-1
*ilr*4c	-1	-1	1	-1	1	1	-1	-1	-1	0	1	-1	-1	-1	-1	-1	0	0	1
*ilr*5c	0	0	-1	0	-1	-1	0	0	0	0	1	0	0	0	0	0	0	0	-1
*ilr*6c	0	0	1	0	-1	-1	0	0	0	0	0	0	0	0	0	0	0	0	1
*ilr*7c	0	0	1	0	0	0	0	0	0	0	0	0	0	0	0	0	0	0	-1
*ilr*8c	0	0	0	0	1	-1	0	0	0	0	0	0	0	0	0	0	0	0	0
*ilr*9c	-1	-1	0	-1	0	0	-1	-1	-1	0	0	-1	-1	-1	-1	1	0	0	0
*ilr*10c	-1	-1	0	-1	0	0	1	1	1	0	0	-1	-1	1	-1	0	0	0	0
*ilr*11c	0	0	0	0	0	0	1	1	-1	0	0	0	0	-1	0	0	0	0	0
*ilr*12c	0	0	0	0	0	0	1	-1	0	0	0	0	0	0	0	0	0	0	0
*ilr*13c	0	0	0	0	0	0	0	0	1	0	0	0	0	-1	0	0	0	0	0
*ilr*14c	-1	1	0	-1	0	0	0	0	0	0	0	-1	-1	0	-1	0	0	0	0
*ilr*15c	1	0	0	-1	0	0	0	0	0	0	0	-1	-1	0	-1	0	0	0	0
*ilr*16c	0	0	0	-1	0	0	0	0	0	0	0	-1	-1	0	1	0	0	0	0
*ilr*17c	0	0	0	-1	0	0	0	0	0	0	0	-1	1	0	0	0	0	0	0
*ilr*18c	0	0	0	1	0	0	0	0	0	0	0	-1	0	0	0	0	0	0	0

Note: 1. -1. and 0 means presence of the corresponding fatty acid in the numerator. denominator. or omitted. respectively

Table S5. *Ilr coordinates* obtained by SBP. based on Ward's clustering sequence

Processing phases	*ilr*1c	*ilr*2c	*ilr*3c	*ilr*4c	*ilr*5c	*ilr*6c	*ilr*7c	*ilr*8c	*ilr*9c
MT0	-2.74270	-3.72421	-2.62670	2.33660	0.71698	-0.94121	5.06299	1.36621	1.21610
MT0	-3.25549	-4.02634	-3.05802	2.27006	0.65812	-1.03594	5.05448	1.31613	1.18395
MT1	-3.10450	-4.16806	-3.14832	2.30033	0.62778	-1.03878	5.03394	1.31035	1.16401
MT1	-3.24550	-4.09542	-3.09853	2.29408	0.64336	-1.07407	5.07967	1.30955	1.17428
MT1	-3.26291	-4.17173	-3.21474	2.29247	0.66087	-1.05467	5.07984	1.30648	1.16651
MT1	-2.90271	-4.03029	-3.04711	2.30738	0.65139	-1.02945	5.04135	1.30385	1.16793
MT2	-3.30285	-3.32874	-2.84563	2.42410	0.71279	-0.86693	5.00272	1.34119	1.26583
MT2	-2.95906	-4.90026	-2.28823	2.34065	0.67585	-0.97813	5.03857	1.29662	1.23404
MT2	-3.07908	-4.04687	-2.23684	2.32149	0.64139	-1.02502	5.04266	1.29688	1.21385
MT2	-3.10937	-4.87858	-2.77697	2.31486	0.64853	-1.03455	5.05867	1.30021	1.21569
MT3	-3.23237	-3.51608	-3.03814	2.36603	0.69983	-0.92545	5.03932	1.32123	1.24720
MT3	-3.08087	-4.07666	-3.20883	2.32972	0.67083	-0.97572	5.03563	1.29333	1.23015
MT3	-3.03853	-4.04946	-3.20904	2.29286	0.63772	-1.07113	5.09267	1.29663	1.22022
MT3	-3.01956	-4.84940	-3.18638	2.28791	0.64185	-1.07836	5.10453	1.29800	1.21874
HT0	-2.47091	-3.87444	-2.80084	1.86227	0.40264	-1.00409	5.12823	1.25306	1.50738
HT0	-2.54712	-4.00170	-3.02724	1.81067	0.37868	-1.00671	5.09052	1.23905	1.47070
HT1	-2.49831	-3.82233	-2.63766	1.81801	0.43285	-0.99662	5.12297	1.21950	1.54330
HT1	-2.47513	-4.03438	-2.91108	1.81534	0.42863	-0.99724	5.12135	1.21980	1.53451
HT1	-2.48599	-4.84276	-2.82815	1.85825	0.42315	-0.98303	5.07564	1.23966	1.48174
HT1	-2.54580	-3.92736	-2.99326	1.85979	0.41469	-0.97071	5.05026	1.23516	1.47768
HT2	-2.39847	-3.72276	-3.11298	1.89739	0.48732	-0.89768	5.10394	1.26931	1.54670
HT2	-4.76107	-3.83138	-3.18159	1.87740	0.43613	-0.93609	5.06148	1.23450	1.51195
HT2	-2.54311	-3.81999	-2.49391	1.83862	0.42145	-0.96458	5.06172	1.22330	1.51265
HT2	-2.55282	-3.97141	-2.31338	1.83499	0.41806	-0.96363	5.06625	1.22990	1.49606
HT3	-2.53199	-3.83121	-2.97882	1.83038	0.43931	-0.95634	5.07998	1.22532	1.51054
HT3	-2.51539	-3.93069	-3.01272	1.83965	0.43153	-0.94789	5.06619	1.22533	1.50967
HT3	-2.21822	-3.90931	-3.26144	1.78910	0.44610	-0.98348	5.11783	1.23287	1.49048
HT3	-2.45901	-3.78438	-3.10140	1.84927	0.41441	-0.93804	5.03877	1.22463	1.51003

Notes: M. Manzanilla; H. Hojiblanca; T0. fresh olives; T1. lye-treated olives; T2. fermented olives; T3. packaged olives.

Table S5. *Ilr coordinates* obtained by SBP. based on Ward's clustering sequence (cont)

Processing phases	*ilr*10c	*ilr*11c	*ilr*12c	*ilr*13c	*ilr*14c	*ilr*15c	*ilr*16c	*ilr*17c	*ilr*18c
MT0	-3.04866	-1.18568	-1.47458	-1.03564	-3.33874	-4.30642	1.18658	4.64914	-0.42316
MT0	-2.67608	-1.11759	-1.42300	-0.94634	-3.69923	-4.22844	1.19256	4.66611	-0.43058
MT1	-2.56308	-1.14585	-1.44845	-0.89860	-3.66641	-4.39676	1.10311	4.65802	-0.41786
MT1	-2.57817	-1.18656	-1.51148	-0.89146	-3.67617	-4.41478	1.10464	4.65958	-0.41314
MT1	-2.58200	-1.18208	-1.49523	-0.88831	-3.66115	-4.40691	1.13016	4.66439	-0.42060
MT1	-2.60384	-1.19450	-1.52430	-0.88754	-3.65409	-4.37605	1.13595	4.66653	-0.42361
MT2	-2.89617	-1.13754	-1.44127	-1.03813	-3.69223	-4.32480	1.21305	4.65022	-0.42049
MT2	-2.61559	-1.14301	-1.47822	-0.92977	-3.70262	-4.41416	1.23812	4.68726	-0.39851
MT2	-2.56547	-1.13158	-1.46902	-0.88943	-3.68404	-4.38472	1.19670	4.68990	-0.39834
MT2	-2.57203	-1.14358	-1.50018	-0.88195	-3.70638	-4.38682	1.19047	4.68067	-0.40806
MT3	-2.76320	-1.12157	-1.54198	-0.98899	-3.67173	-4.38941	1.20193	4.67023	-0.44393
MT3	-2.60802	-1.16735	-1.49820	-0.90748	-3.70714	-4.40419	1.21207	4.68700	-0.42467
MT3	-2.54746	-1.14185	-1.50233	-0.87837	-3.68132	-4.38580	1.14094	4.68453	-0.39277
MT3	-2.53999	-1.14971	-1.50294	-0.86603	-3.69352	-4.38983	1.13879	4.68242	-0.40209
HT0	-2.60283	-1.31224	-1.43609	-1.00413	-3.72104	-4.36254	1.10989	4.73368	-0.40682
HT0	-2.58529	-1.33485	-1.44942	-0.96940	-3.51534	-4.36936	1.10979	4.73614	-0.42034
HT1	-2.52794	-1.30051	-1.44823	-0.97044	-3.74307	-4.32030	1.15338	4.73324	-0.43505
HT1	-2.50364	-1.31798	-1.45869	-0.94237	-3.72889	-4.33936	1.15104	4.73419	-0.43179
HT1	-2.55272	-1.29185	-1.46971	-0.93538	-3.75129	-4.33350	1.12272	4.69345	-0.42009
HT1	-2.53283	-1.30497	-1.45671	-0.91752	-3.72701	-4.35062	1.12333	4.69748	-0.41880
HT2	-2.80924	-1.27849	-1.50158	-1.03749	-3.72856	-4.25217	1.18497	4.68070	-0.38627
HT2	-2.58826	-1.28342	-1.49327	-0.95342	-3.74844	-4.28255	1.19917	4.71331	-0.41847
HT2	-2.54824	-1.27741	-1.46290	-0.92655	-3.75652	-4.27118	1.17985	4.70353	-0.42853
HT2	-2.53468	-1.29160	-1.48058	-0.90392	-3.73623	-4.25235	1.17777	4.70953	-0.40898
HT3	-2.53150	-1.25199	-1.46717	-0.91739	-3.72013	-4.28979	1.21871	4.71577	-0.42035
HT3	-2.54113	-1.25375	-1.47025	-0.92630	-3.73225	-4.27526	1.21665	4.71625	-0.42388
HT3	-2.50172	-1.22861	-1.36337	-0.89642	-3.73992	-4.26475	1.20056	4.71329	-0.41670
HT3	-2.51914	-1.25526	-1.42822	-0.90889	-3.74487	-4.30011	1.20053	4.71149	-0.42507

Notes: M. Manzanilla; H. Hojiblanca; T0. fresh olives; T1. lye-treated olives; T2. fermented olives; T3. packaged olives. Treatments within cultivar are formed by combining cultivar and processing phases.

Table S6. Means and variances of the balances obtained by SBP based on the decreasing *clr* variance and Ward's clustering sequence

	Decreasing *clr* variance		Ward's clustering	
	Mean	Variance	Mean	Variance
Balance 1	-2.8692	0.2389	-2.8692	0.2389
Balance 2	-4.0416	0.1459	-4.0416	0.1459
Balance 3	-2.9156	0.0847	-2.9156	0.0847
Balance 4	1.3004	0.0414	2.0807	0.0583
Balance 5	1.064	0.0315	0.5451	0.0146
Balance 6	2.2841	0.0112	-0.9884	0.0027
Balance 7	-1.264	0.0108	5.0697	0.001
Balance 8	-3.3464	0.0068	1.2724	0.0018
Balance 9	-3.3994	0.0087	1.3579	0.0231
Balance 10	-0.1537	0.0043	-2.6085	0.0152
Balance 11	-3.7616	0.0037	-1.219	0.005
Balance 12	3.0905	0.0044	-1.4713	0.0013
Balance 13	-3.726	0.0036	-0.9335	0.0024
Balance 14	-1.8867	0.0011	-3.6903	0.0067
Balance 15	-2.7005	0.0037	-4.3383	0.0032
Balance 16	-2.4026	0.0014	1.1691	0.0016
Balance 17	3.2796	0.0005	4.6924	0.0007
Balance 18	-2.1712	0.0045	-0.4171	0.0002

Table S7. Proportions of successful assignation after application of Linear Discriminant Analysis. using bootstrapping (1000). to the *ilr coordinates* based on the *clr* variance decreasing order and Ward's clustering sequences

From/to	MTO	MT1	MT2	MT3	HT0	HT1	HT2	HT3
MT0	100.100							
	(50. 0)		(50. 100)					
MT1		100.100						
		(50. 100)		(50. 0)				
MT2			100.100					
	(25. 0)		(75.75)	(0. 25)				
MT3				100. 100				
		(50. 0)		(50. 100)				
HT0					100. 100			
					(50. 100)	(50. 0)		
HT1						100. 100		
						(100. 100)		
HT2							100.100	
						(50. 0)	(25.25)	(25.75)
HT3								100.100
							(25.0)	(75. 100)

Notes: Results of one-out validation (based on *clr* variance and Wards' clustering. respectively) below in bracket. M. Manzanilla; H. Hojiblanca; T0. fresh olives; T1. lye-treated olives; T2. fermented olives; T3. packaged olives.
